# Magnetofection-mediated siRNA delivery ameliorates cartilage damage in Kashin-Beck disease *via* targeting SIRT3 and protecting primary cilia

**DOI:** 10.7150/thno.134335

**Published:** 2026-07-22

**Authors:** Yangmengfan Chen, Duan Wang, Yao Zhang, Xiaoyang Liu, Ze Du, Xuming Chen, Xufeng Wan, Yongrui Cai, Anjing Chen, Jiehao Chen, Hao Du, Zongke Zhou

**Affiliations:** 1 Department of Orthopedics and Research Institute of Orthopedics, West China Hospital, Sichuan University, Chengdu, 610041, China.; 2 Animal Experimental Center, West China Hospital Sichuan University, Chengdu, 610041, China.

**Keywords:** Kashin-Beck disease, electromagnetic field, biomaterial, T-2 toxin, molecular mechanism

## Abstract

**Rationale:**

Kashin–Beck disease (KBD) is an endemic osteochondropathy caused by T-2 toxin, however, the molecular mechanism underlying T-2 toxin–induced chondrocyte damage remains unclear. This study aimed to elucidate the pathogenic role of T-2 toxin in KBD and develop an EMF-augmented nanotherapy for KBD-related cartilage damage.

**Methods:**

This study investigated T-2 toxin-induced chondrocyte damage by evaluating protein acetylation, primary cilia integrity and the levels of chondrogenic markers (Sox9, Col2a1). *In vitro* experiments were performed via pharmacological and genetic SIRT3 inhibition. To protect chondrocytes, si-*SIRT3*@SPIONs were fabricated, and EMF was applied to improve cell transfection and silencing efficiency. A KBD model in SD rats was used to validate the *in vivo* therapeutic effect; immunohistochemical staining and micro-CT scanning and reconstruction were performed to comprehensively evaluate* in vivo* treatment efficacy.

**Results:**

Pathological *SIRT3* overexpression induced by T-2 toxin disrupted chondrocyte protein acetylation, impaired primary cilia integrity, and suppressed the gene expression of chondrogenic markers. *SIRT3* inhibition efficiently protected T-2 toxin-induced chondrocyte cytotoxicity* in vitro*. Furthermore, EMF-augmented si-*SIRT3*@SPIONs treatment ameliorated cartilage damage, preserved matrix composition and restored normal chondrocyte phenotype in KBD model rats.

**Conclusions:**

T-2 toxin induces chondrocyte injury and promotes KBD progression mainly by disrupting primary cilia integrity and protein acetylation, with *SIRT3* overexpression acting as a mediating factor. The EMF-augmented si-*SIRT3*@SPIONs therapy can effectively protect T-2 toxin-induced primary cilia damage and cartilage degeneration, thus providing a promising therapeutic modality for KBD.

## Introduction

Kashin-Beck disease (KBD) is a chronic and degenerative osteochondropathy, which is endemic to a limited geographic region stretching from northeastern to southwestern China, and also occurs in certain parts of Mongolia, Siberia, and North Korea 1. In the early stages, KBD patients typically present with shortened and enlarged fingers. As KBD progresses, advanced cases may exhibit deformities of the limb joints, restricted mobility, and even dwarfism 2. According to the 2013 Chinese Health Statistics, approximately 38.07 million people live in KBD-prevalent areas. KBD affects a total of 644,994 individuals in these endemic regions 3. Among these cases, 16,826 are children under 13 years of age 4. The Chinese government's long-term prevention and control programs have significantly reduced the overall incidence of KBD, yet new cases still emerge, particularly in western China 5.

T-2 toxin, a type A trichothecene mycotoxin, is frequently detected in foodstuffs, animal feed, and agricultural products in KBD prevalent areas 6. T-2 toxin is produced primarily by *Fusarium* species and other fungal genera, and has been identified in various field crops (barley, maize, wheat, and oats) and processed grain products (beer, malt, and bread) 7. Our skeletal system is the primary target harmed by T-2 toxin 8. It has been reported that T-2 toxin can induce chondrocyte apoptosis 9, by up-regulating apoptosis-related genes 10. In patients with KBD, chondrocyte necrosis occurs primarily in the deep zone of the cartilage 11, thus the cartilage erosion within the hypertrophic layer represents a fundamental pathological characteristic of KBD, which differs from osteoarthritis (OA) 12. In addition, chondrocyte injury in KBD also involves dysregulation of metabolic processes, immune responses, cytoskeletal alterations, and extracellular matrix (ECM) synthesis 13. However, the underlying molecular mechanisms lead to the pathogenesis and progression of KBD remain poorly understood 14. Therefore, effective therapeutic strategies for KBD remain limited.

Recently, accumulating evidence indicates that cartilage damage in KBD results from the interplay of genetic and environmental factors 15. A relevant study performed genome-wide DNA methylation profiling of articular cartilage and revealed widespread epigenetic dysregulation involving multiple genes in KBD 16, and the regulation of epigenetic mechanisms can delay the progression of joint damage 17. Sirtuins (SIRTs), a family of mitochondrial deacetylases, regulate cellular metabolism by deacetylating key enzymes and modulating mitochondrial proteostasis 18. However, the disruption of proteostatic balance can also lead to this tissue damage. For instance, the overexpression of *SIRT3* has been reported to disrupt proteostatic balance, resulting in excessive ROS generation, cell cycle arrest 19, autophagy, and ferroptosis 20. And the overexpression of other SIRT members, such as SIRT1 21 and SIRT2 22 impaired the formation of primary cilia by inducing degradation of ciliary proteins. The damage of primary cilia attenuates cells’ ability to sense extracellular physiochemical stimuli 23, and eventually impairs chondrocytes differentiation 24. In this study, transcriptome sequencing screening revealed that *SIRT3* is remarkably overexpressed after T-2 toxin treatment, which provides a potential therapeutic target for treating KBD.

RNA interference is a well-established gene silencing strategy with approval from the FDA for certain therapeutic applications 25. However, effective siRNA delivery to chondrocytes remains challenging due to the avascular structure and dense ECM of cartilage tissue 26. Furthermore, the inherent instability of siRNA molecules often leads to rapid clearance from the synovial fluid and degradation by nucleases within hours 27. To solve this challenge, siRNA-based nanotherapies have gained increasing attention as a promising strategy for targeted delivery and gene silencing in recent decades 28. For instance, a pioneering work proposed a multifunctional siRNA-nanoparticles (NPs) with the ability of specific targeting, siRNA loading and protection, and pH-triggered cytosolic release, therefore effectively silencing *PHB1* and inhibiting tumor growth 29. To further improve the safety and precision, a near-infrared (NIR) imaging-guided siRNA nanoplatform has been fabricated. Shi *et al.* coated NIR fluorescent polymeric NP (~50 nm) in siRNA nanocomplex to achieve a non-invasive NIR imaging, precise guiding, and efficient target gene silencing in specific tissue 30. Considering that the transfection efficiency of siRNA is a critical determinant of gene therapy efficacy 31, especially within the unique microenvironment of the articular cavity, we developed a novel strategy to improve it in this study. Electromagnetic fields (EMFs) are regarded as a safe, non-invasive, and effective modality, with promising therapeutic potential for a variety of diseases 32. Moreover, EMF has excellent penetration capabilities, enabling them to traverse the skin, adipose tissue, and fascial layers to reach deep tissues 33. To improve the transfection efficiency in the articular cavity, in this study, si-*SIRT3* was conjugated to the surface of SiO_2_-coated superparamagnetic iron oxide nanoparticle (si-*SIRT3*@SPION). Upon the application of an electromagnetic field (EMF), SPIONs can be guided into close proximity to target cells via magnetic forces 34, exhibited high cellular uptake efficiency and minimal cytotoxicity 35. This approach may represent a novel therapeutic strategy for KBD.

This study explored the toxicological effects of T-2 toxin on chondrocyte viability, function, and primary cilia integrity. We further explored the transcriptional and epigenetic alterations associated with KBD pathogenesis and identified *SIRT3* as a key regulator in this process. Subsequently, we developed si-*SIRT3*@SPION in combination with EMF exposure as a novel targeted therapeutic strategy to mitigate KBD-related cartilage damage. Collectively, our findings not only elucidate critical mechanisms underlying KBD progression but also offer a promising therapeutic approach. Moreover, this study establishes a research and treatment paradigm potentially applicable to other endemic diseases.

## Results

### KBD clinical specimens exhibit an inside-out damage to articular chondrocytes

**Figure 1:**
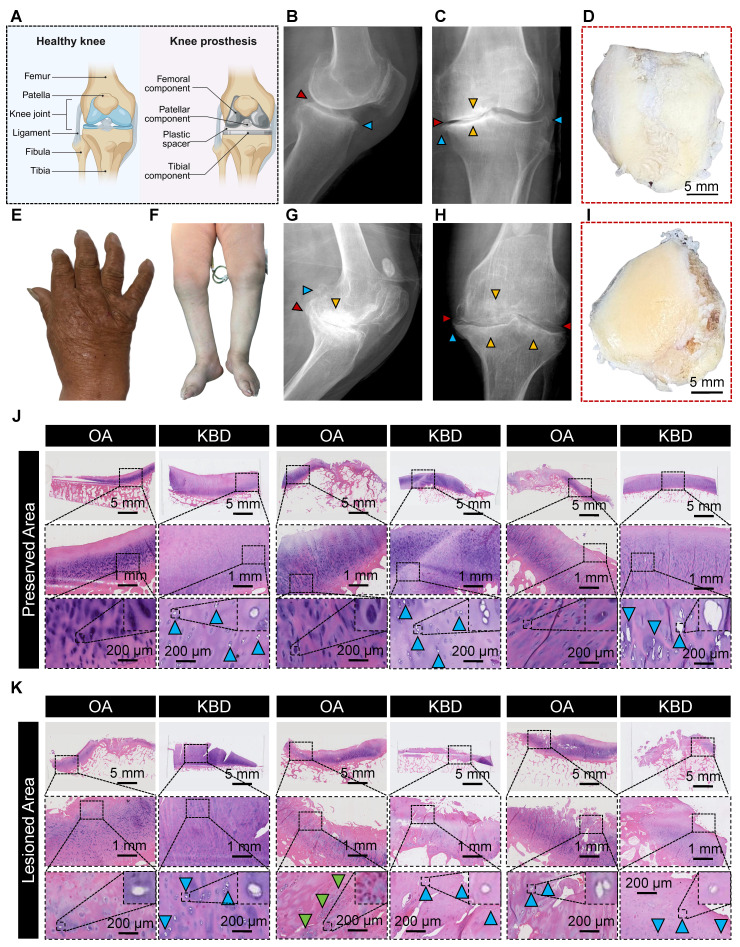
** Histological features of KBD and OA. (A)** Illustrative scheme of total knee arthroplasty. **(B, C)** Anteroposterior and lateral view radiographs of the knee of OA patient (red arrows indicate joint space narrowing, blue arrows indicate osteophyte formation, and yellow arrows indicate subchondral sclerosis). **(D)** Gross view of the resected of cartilage in OA patient. **(E)** Brachydactyly and enlargement/deformity of interphalangeal joints and wrist in KBD patient. **(F)** Deformity of knee and ankle in KBD patient. **(G, H)** Anteroposterior and lateral view radiographs of the knee of KBD patient (red arrows indicate joint space narrowing, blue arrows indicate osteophyte formation, and yellow arrows indicate subchondral sclerosis). **(I)** Gross view of the resected of cartilage in KBD patient. H&E staining of **(J)** preserved area, and (K) lesioned area of cartilage from OA and KBD patients. N ≥ 3, n = 3.

Total knee arthroplasty (TKA) is considered as a most effective surgery for the end-stage OA and KBD patients with knee joint damage. TKA can replace the damaged knee joint surfaces with metal components and plastic spacer to restore normal alignment and function of knee joint. To investigate the pathological alterations of KBD, we performed histological analyses on tibial plateau cartilage specimens obtained from patients undergoing TKA for KBD or OA patients **(Figure 1A)**. As the radiographic features of OA showed, the osteophyte formed around the joint (blue arrows) as the degradation. Importantly, the knee joint space narrowing (red arrows), and the subchondral sclerosis (yellow arrows) usually occur in the medial compartment, rather than lateral compartment, due to weight-bearing stress **(Figure 1B-D)**. In contrast, KBD patients exhibited as brachydactyly and enlargement of interphalangeal joints **(Figure 1E)** and the deformity of ankle joints **(Figure 1F)**. Of note, the radiographic features and gross appearance of cartilage destruction of knee joint of KBD patients **(Figure 1 G-I)** differ with OA. In KBD, the knee joint space narrowing (red arrows) and subchondral sclerosis (yellow arrows) are more severe and extensive to both compartments, and it also shows the features of osteoporosis. These different features demonstrated remarkably differences between OA and KBD. Briefly, OA is considered as the degenerative disease mainly caused by aging and mechanical force. While, KBD is a widespread and pathological destruction of bone and cartilage.

The histological evaluation revealed pronounced differences between OA and KBD, which implies the different pathological mechanisms. In the preserved area, where are non-weight-bearing zone in the knee joints, the surfaces of cartilage are smooth and the morphology of chondrocytes is intact. In contrast, there is a significant increase in empty chondrocyte lacunae (blue arrows) in KBD specimens** (Figure 1J)**. In the lesioned area, where are weight bear zone, the surfaces of cartilage are obviously destructed in both OA and KBD specimens. Interestingly, there are empty chondrocyte lacunae (blue arrows) and extensive chondrocyte clustering (green arrows) existed in the superficial zone of lesion area in OA cartilage **(Figure 1K)**. However, the immunofluorescent staining for HIF-1α **(Figure S1A)** and VEGF **(Figure S1B)** showed no obvious differences. In summary, KBD is an endemic and developmental osteochondropathy, the cartilage degradation in KBD follows a pathognomonic inside-to-outside pattern, indicated by the existence of empty chondrocyte lacunae in both superficial and deep cartilage zones. In contrast, OA is considered as a degenerative joint disorder that caused by aging and mechanical force, typically presenting with localized cartilage degradation, empty chondrocyte lacunae, and chondrocyte clustering.

### T-2 toxin compromises chondrocyte viability and up-regulates *SIRT3* expression

**Figure 2:**
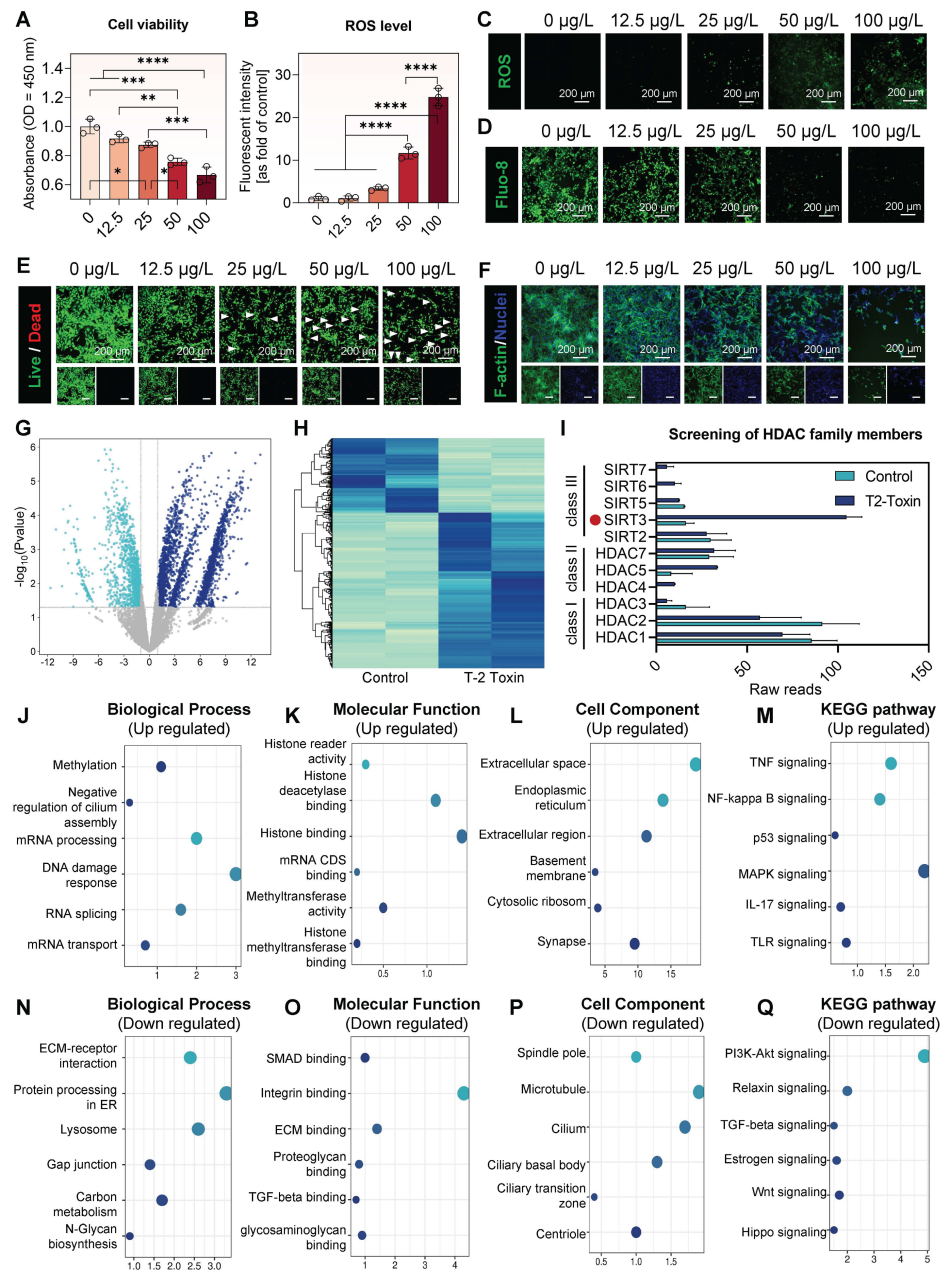
** Cytotoxic effects of T-2 toxin on chondrocytes and transcriptomic profiling. (A)** Viability of chondrocytes treated with different concentrations of T-2 toxin. **(B, C)** Quantitative analysis and representative fluorescence images of intracellular ROS levels in chondrocytes after T-2 toxin treatment. **(D)** Representative fluorescence images of intracellular Ca^2+^ levels detected using Fluo-8 AM. **(E)** Live/dead staining showing viability of chondrocytes treated with T-2 toxin. **(F)** Cytoskeletal organization (phalloidin, green) and nuclei (DAPI, blue) in chondrocytes following T-2 toxin treatment. **(G)** Volcano plot of differentially expressed genes. **(H)** Heatmap of general gene expression profiles. **(I)** Expression levels of HDAC family members, highlighting *SIRT3* as the most significantly altered gene. **(J - M)** Significantly enriched up-regulated GO terms and KEGG pathways. **(N - Q)** Significantly enriched down-regulated GO terms and KEGG pathways. Statistical significance was calculated using one-way ANOVA with Tukey’s multiple comparisons test: * p < 0.05, *** p < 0.001, **** p < 0.0001. N=3, n=3.

Since T-2 toxin is recognized as a major etiological factor in KBD, we initially assessed its cytotoxic effects on chondrocytes. Cell viability assays revealed that T-2 toxin induced a significant reduction in cell viability in a dose-dependent manner **(Figure 2A)**. Concurrently, T-2 toxin exposure markedly elevated intracellular ROS levels, particularly at concentrations of 50 μg/L and 100 μg/L **(Figure 2B, C)**. The oxidative stress led to a decrease in intracellular Ca^2+^ concentration, which is a critical second messenger involved in numerous signaling pathways and the maintenance of cellular homeostasis **(Figure 2D)**. Consistent with the viability results, live/dead staining further verified the cytotoxicity of T-2 toxin, demonstrating a decline in live cells (green) and an increase in dead cells (red) with increased toxin concentrations **(Figure 2E)**. Moreover, staining of the cytoskeleton with phalloidin (green) and nuclei with DAPI (blue) revealed impaired cell spreading and structural disruption of the actin cytoskeleton following T-2 toxin treatment **(Figure 2F)**. Based on the significant effects of T-2 toxin at 50 μg/L on various cellular processes, this concentration was selected for subsequent experiments in the study.

To clarify the molecular mechanisms regarding the cytotoxic effects of T-2 toxin on chondrocytes, we performed transcriptome sequencing analysis. The volcano plot revealed 1726 significantly up-regulated and 696 significantly down-regulated genes following T-2 toxin treatment **(Figure 2G)**. Consistent with these findings, the heatmap further demonstrated substantial alterations in the transcriptomic profile of chondrocytes induced by T-2 toxin **(Figure 2H)**. Subsequent in-depth analysis of the sequencing data highlighted the histone deacetylase (HDAC) family as being notably affected. Among these, *SIRT3* emerged as the most differentially expressed gene, and exhibited the most significant changes **(Figure 2I)**, suggesting a critical role of *SIRT3* in the chondrocyte response to T-2 toxin treatment.

To investigate the functional impact of T-2 toxin-induced transcriptional changes, we performed Gene Ontology (GO) and Kyoto Encyclopedia of Genes and Genomes (KEGG) enrichment analyses. Among the up-regulated GO terms, epigenetic regulation and negative regulation of primary cilia were significantly enriched **(Figure 2J–L)**. KEGG analysis of up-regulated pathways revealed enrichment in multiple inflammatory signaling pathways **(Figure 2M)**. Conversely, down-regulated GO terms were associated with ECM activities and primary cilium-related cellular components **(Figure 2N-P)**. And the down-regulated KEGG pathways included several protective signaling pathways **(Figure 2Q)**. Collectively, these results demonstrate that T-2 toxin exerts detrimental effects on chondrocytes by promoting ROS generation, altering epigenetic regulation, and disrupting primary cilium. Notably, *SIRT3* was identified as the most significantly altered target, suggesting a critical role in KBD.

### T-2 toxin impairs acetylation, primary cilia integrity, and chondrogenic capacity

**Figure 3:**
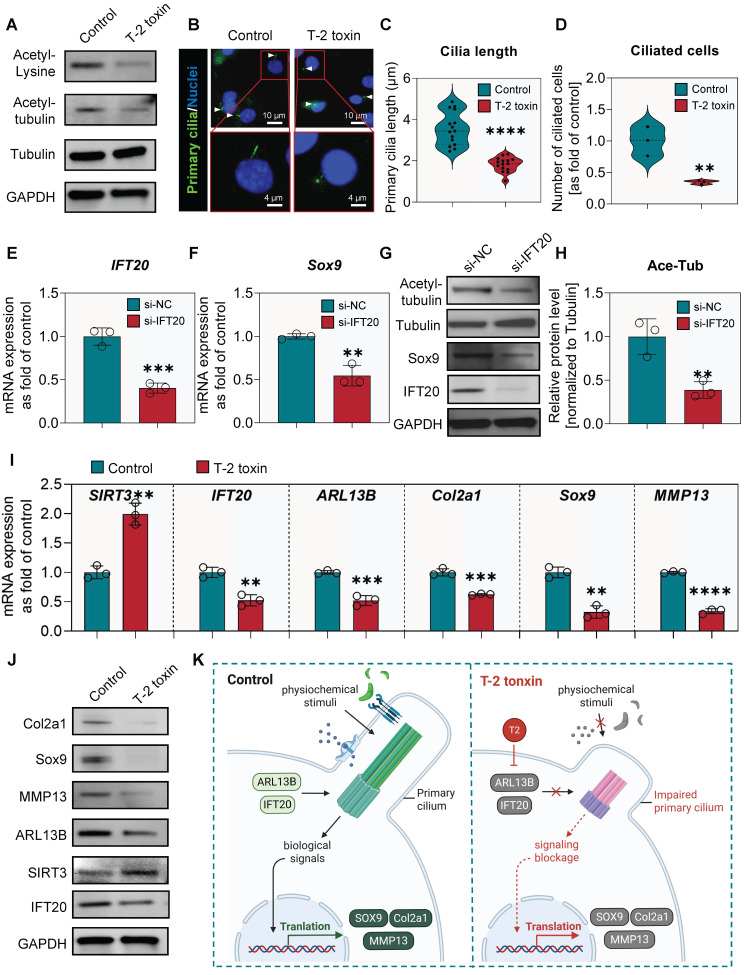
** T-2 toxin impairs chondrocyte function by disrupting protein acetylation, primary cilium integrity, and chondrogenic capacity. (A)** Western blot of acetyl-Lysine, acetyl-tubulin, total tubulin, and GAPDH in chondrocytes treated with T-2 toxin. **(B)** Representative immunofluorescence images of primary cilia (acetylated-tubulin, green) and nuclei (DAPI, blue). **(C)** Quantitative analysis of the length of primary cilia, and **(D)** the proportion of ciliated chondrocytes, compared with the control. The mRNA expression of **(E)** *IFT20*, and **(F)** *Sox9* in chondrocytes treated with or without si-*SIRT3*, compared with the control. **(G)** Protein levels of acetyl-Lysine, acetyl-tubulin, tubulin, and GAPDH in chondrocytes treated with or without si-*SIRT3*. **(H)** Quantification of acetyl-tubulin protein levels normalized to total tubulin, compared with the control. **(I)** RT-qPCR analysis of *SIRT3*, *IFT20*, *ARL13B*, *Col2a1*, *Sox9*, and *MMP13* in chondrocytes, compared with the control. **(J)** Western blot of SIRT3, IFT20, ARL13B, Col2a1, Sox9, and MMP13 in chondrocytes. **(K)** Schematic illustration of the toxic influences of T-2 toxin on chondrocytes. Statistical significance was calculated using two-tailed t-test: ** p < 0.01, *** p < 0.001, **** p < 0.0001. N=3, n=3.

Due to the observed alterations in HDAC family members, we next assessed the intracellular protein acetylation levels. Western blot analysis revealed that T-2 toxin treatment significantly reduced the levels of acetyl-Lysine and acetylated-tubulin in chondrocytes **(Figure 3A)**. Since acetylated-tubulin is a major structural component of the primary cilium, we further examined cilium integrity by immunofluorescence staining **(Figure 3B)**. Quantitative analysis demonstrated that T-2 toxin severely disrupted primary cilia, manifesting as significant shortening of cilia length **(Figure 3C)** and a reduced proportion of ciliated chondrocytes **(Figure 3D)**. Primary cilia are critical sensory organelles that transduce physicochemical signals from the extracellular microenvironment into biological responses to regulate cellular processes. To investigate the functional importance of primary cilia in chondrocytes, we knocked down intraflagellar transport 20 (*IFT20*) using siRNA **(Figure 3E)**. *IFT20* KD resulted in loss of primary cilia and subsequently downregulated the expression of the chondrogenic marker *Sox9* **(Figure 3F)**. Consistent with these findings, western blot analysis verified that *IFT20* KD led to reduced protein levels of IFT20, Sox9 **(Figure 3G, Figure S2)**, and acetylated-tubulin **(Figure 3H)**. Together, these results indicate that T-2 toxin induces hypoacetylation of cellular proteins and disrupts primary cilium assembly and maintenance. Furthermore, cilium loss may compromise the chondrogenic potential of chondrocytes, likely through impaired *Sox9* expression.

To further verify the mechanism by which T-2 toxin influences chondrocyte fate, the RT-qPCR was conducted to evaluate several key genes’ expression. The results demonstrated that T-2 toxin significantly up-regulated *SIRT3* expression, consistent with our transcriptome sequencing data. Notably, the expression of genes encoding core ciliary components, *IFT20* and *ARL13B*, was markedly reduced following T-2 toxin treatment. Concomitantly, the expression of *Sox9* and *MMP13* was also down-regulated, indicating impaired chondrogenic capacity and dysregulated matrix metabolism in chondrocytes treated with T-2 toxin **(Figure 3I)**. These transcriptional changes were further verified at the protein level, confirming the toxicological impact of T-2 toxin **(Figure 3J, Figure S3).**

As summarized in the schematic illustration **(Figure 3K)**, T-2 toxin downregulates ARL13B and IFT20 expression, leading to the impaired primary cilia integrity. The impaired primary cilia limited chondrocyte ability to perceive extracellular physiochemical stimuli, therefore decreasing the chondrogenic capacity and the regeneration of cartilage.

### Pharmacological inhibition of *SIRT3* attenuates T-2 toxin-induced chondrocyte injury

**Figure 4:**
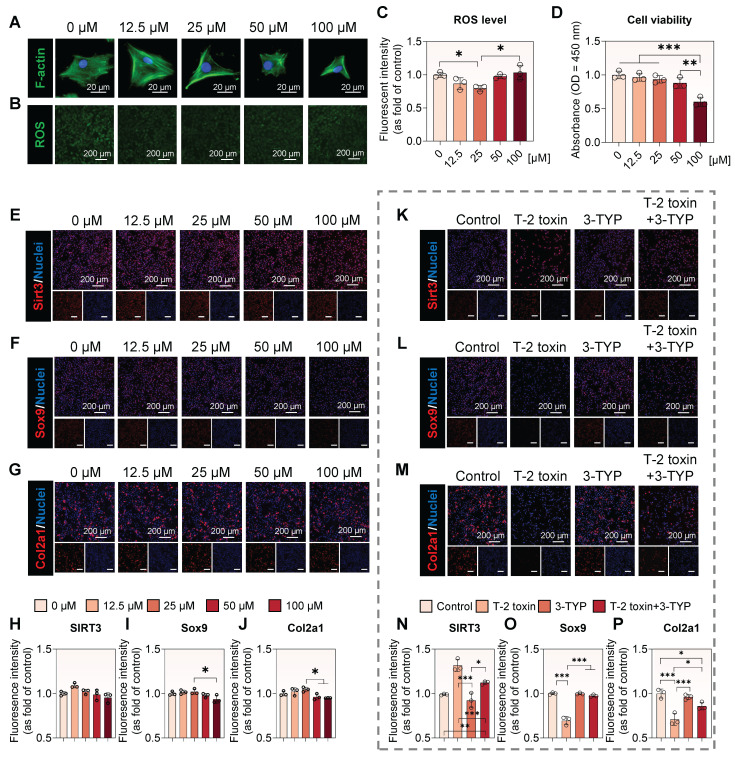
** Inhibition of SIRT3 with 3-TYP protect chondrocytes from the damage caused by T-2 toxin. (A)** Cytoskeleton (phalloidin, green) and nuclei (DAPI, blue) in chondrocytes treated with different concentrations of 3-TYP. **(B, C)** Representative fluorescence images and the quantitative analysis of intracellular ROS levels in chondrocytes. **(D)** Cell viability of chondrocytes treated with 3-TYP. Immunofluorescent staining of **(E)** SIRT3, **(F)** Sox9, and **(G)** Col2a1 in chondrocytes treated with different concentrations of 3-TYP. **(H-J)** Quantitative analysis of SIRT3, Sox9, and Col2a1 treated with 3-TYP. Immunofluorescent staining of **(K)** SIRT3, **(L)** Sox9, and **(M)** Col2a1 in chondrocytes treated with T-2 toxin and/or 3-TYP. **(N-P)** Quantitative analysis of SIRT3, Sox9, and Col2a1 treated with T-2 toxin and/or 3-TYP. Statistical significance was calculated using one-way ANOVA with Tukey’s multiple comparisons test: * p < 0.05, ** p < 0.01, *** p < 0.001. N=3, n=3.

To investigate the critical role of SIRT3 in the pathogenesis of KBD, we pharmacologically inhibited SIRT3 activity using the specific inhibitor 3-TYP, and systematically evaluated its effects. Cytoskeleton staining revealed that 3-TYP at 12.5 μM and 25 μM showed no obvious effect on cell morphology, whereas 3-TYP at 50 μM and 100 μM induced noticeable cell contraction **(Figure 4A)**. Intracellular ROS staining **(Figure 4B)** and its quantitative analysis **(Figure 4C)** demonstrated that ROS levels were slightly reduced at 3-TYP concentrations below 25 μM but increased significantly at concentrations ≥50 μM. Cell viability assays further indicated that 100 μM 3-TYP markedly reduced chondrocyte viability **(Figure 4D)**. Moreover, the immunofluorescence staining showed that SIRT3 protein levels were not significantly changed across all concentrations of 3-TYP tested (12.5 - 100 μM) **(Figure 4E, H)**. In contrast, Sox9 protein expression was inhibited at 100 μM 3-TYP **(Figure 4F, I)**, and Col2a1 levels were significantly reduced at concentrations as low as 75 μM **(Figure 4G, J)**. Based on these results, 25 μM 3-TYP was used for the next investigation, as its effects on cell viability and levels of functional protein.

We next evaluated the potential protective effects of 3-TYP against T-2 toxin-induced injury in chondrocytes. The immunofluorescence staining of primary cilia indicated that 3-TYP restored the damaged ciliary integrity caused by T-2 toxin **(Figure S4)**. Immunofluorescence staining of SIRT3 revealed that although T-2 toxin exposure reduced overall cell number, it strongly up-regulated SIRT3 protein levels. Notably, co-treatment with 3-TYP effectively restored this SIRT3 overexpression **(Figure 4K, N)**. Furthermore, T-2 toxin significantly suppressed Sox9 level, while the addition of 3-TYP markedly attenuated this reduction **(Figure 4L, O)**. Similarly, 3-TYP treatment also rescued the decline in Col2a1 levels induced by T-2 toxin **(Figure 4M, P)**. Furthermore, the western blot also verified the therapeutic effects of 3-TYP **(Figure S5)**. Together, these findings indicate that pharmacological inhibition of SIRT3 with 3-TYP alleviates T-2 toxin-induced damage and restores the expression of key chondrogenic markers in chondrocytes.

### *SIRT3* knockdown restores acetylation, ciliogenesis, and chondrogenesis in T-2 toxin-treated chondrocytes

**Figure 5:**
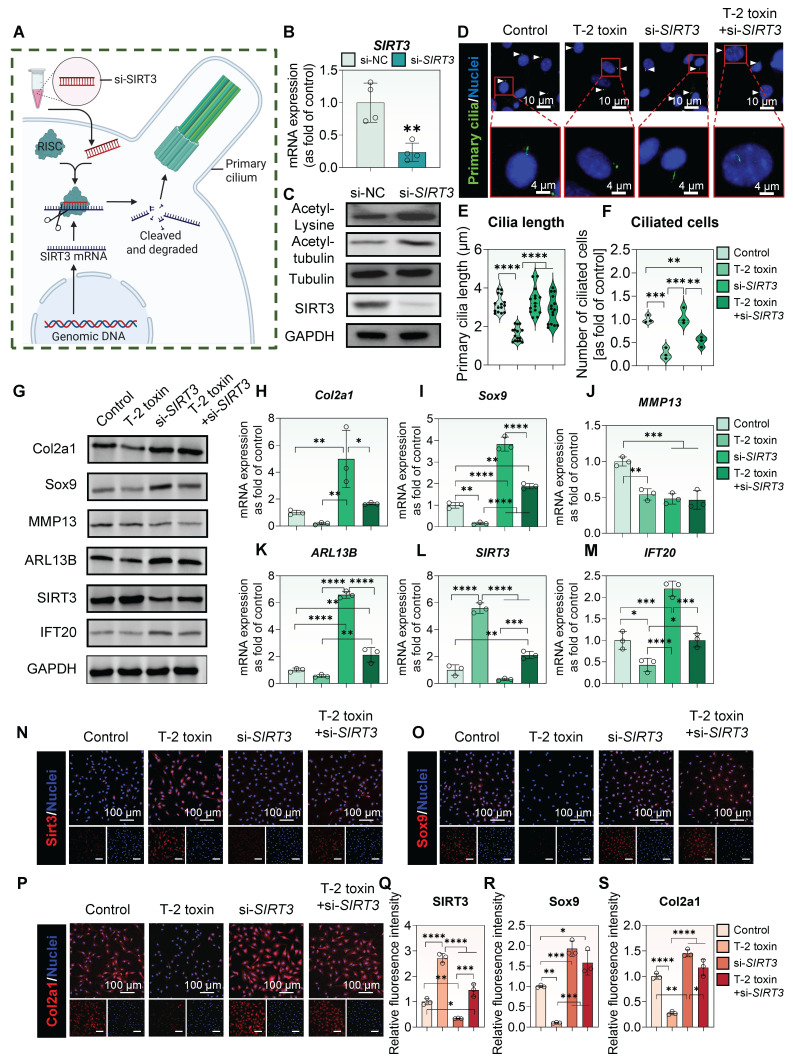
***SIRT3* KD protects chondrocytes from T-2 toxin-induced injury by restoring protein acetylation, primary cilia integrity, and chondrogenic capacity. (A)** Schematic illustration of siRNA-mediated knockdown of *SIRT3*. **(B)** *SIRT3* mRNA expression levels in chondrocytes transfected with si-*SIRT3*. **(C)** Western blot of acetyl-Lysine, acetyl-tubulin, total tubulin, and SIRT3 in chondrocytes after *SIRT3* KD. **(D-F)** Representative fluorescence images and the quantitative analysis of primary cilia in chondrocytes treated with T-2 toxin and/or si-*SIRT3*. **(G)** Western blot of Col2a1, Sox9, MMP13, ARL13B, SIRT3, IFT20, and GAPDH in chondrocytes treated with T-2 toxin and/or si-*SIRT3*. **(H-M)** RT-qPCR analysis of the mRNA expression of *Col2a1*, *Sox9*, *MMP13*, *ARL13B*, *SIRT3*, and *IFT20*. Immunofluorescent staining of **(N)** SIRT3, **(O)** Sox9, and **(P)** Col2a1 in chondrocytes treated with T-2 toxin and/or si-*SIRT3*. **(Q-S)** Quantitative analysis of fluorescence intensity for SIRT3, Sox9, and Col2a1 treated with T-2 toxin and/or si-*SIRT3*. Statistical significance was calculated using two-tailed t-test, or one-way ANOVA with Tukey’s multiple comparisons test: * p < 0.05, ** p < 0.01, *** p < 0.001, **** p < 0.0001.

Given that pharmacological modulation of SIRT3 may lack specificity and potentially affect other HDAC members, we intended to use siRNA-mediated knockdown of *SIRT3* (si-*SIRT3*) to achieve targeted inhibition. With the cleavage and degradation of *SIRT3* mRNA, the effects of overexpression of SIRT3 on ciliary integrity would be blocked **(Figure 5A)**. *In vitro* experiments confirmed that si-*SIRT3* efficiently reduced both mRNA **(Figure 5B)** and protein levels of SIRT3, while increasing the general levels of acetyl-Lysine and acetylated-tubulin **(Figure 5C, Figure S6)**. Next, we evaluated the therapeutic potential of si-*SIRT3* in T-2 toxin-injured chondrocytes. Immunofluorescence analysis of primary cilia **(Figure 5D)** revealed that si-*SIRT3* treatment attenuated T-2 toxin-induced ciliary damage, preserving both cilium length **(Figure 5E)** and the proportion of ciliated cells **(Figure 5F)**. Western blot further demonstrated that *SIRT3* KD restored the expression of multiple key proteins dysregulated by T-2 toxin, including Col2a1, Sox9, MMP13, ARL13B, SIRT3, and IFT20 **(Figure 5G)**. Consistent with these findings, RT-qPCR analysis further verified the recovery of corresponding mRNA levels **(Figure 5H-M)**. Additional immunofluorescence staining validated that si-*SIRT3* reduced SIRT3 protein intensity **(Figure 5N, Q)**, while increasing the expression of Sox9 **(Figure 5O, R)** and Col2a1 **(Figure 5P, S)** in chondrocytes treated with T-2 toxin. Together, these results indicate that targeted knockdown of *SIRT3* represents an effective and precise strategy for counteracting *SIRT3* overexpression in an *in vitro* model of KBD.

### Characterization and enhanced *in vitro* efficacy of si-*SIRT3*@SPIONs under EMF exposure

**Figure 6:**
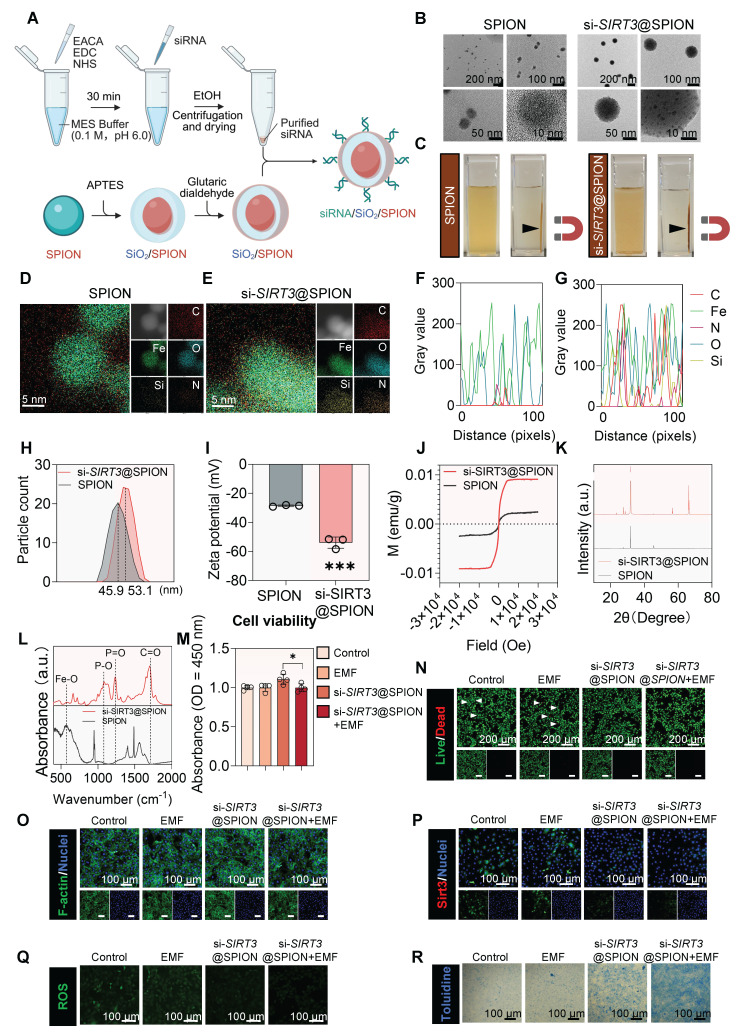
** Characterization of si-*SIRT3*@SPIONs and evaluation of the *in vitro* biocompatibility and chondrogenic effects. (A)** Schematic illustration of the synthesis of si-*SIRT3*@SPIONs. **(B)** TEM images of SPIONs and si-*SIRT3*@SPIONs. **(C)** Paramagnetic behavior of SPIONS and si-*SIRT3*@SPIONs under an external static magnetic field. **(D, E)** Elemental mapping of SPIONS and si-*SIRT3*@SPIONs. **(F, G)** Co-localization analysis of elemental mapping of SPIONS and si-*SIRT3*@SPIONs. **(H)** Particle size distribution analysis of SPIONs and si-*SIRT3*@SPIONs. **(I)** Zeta potential analysis. **(J)** Magnetic hysteresis curves analysis. **(K)** XRD analysis. **(L)** FT-IR spectroscopy of SPIONS and si-*SIRT3*@SPIONs. **(M)** Viability of chondrocytes treated with si-*SIRT3*@SPIONs with/without EMF. **(N)** Live/dead staining of chondrocytes after treatment. **(O)** Cytoskeleton (phalloidin, green) and nuclei (DAPI, blue) of chondrocytes. **(P)** Immunofluorescent staining of SIRT3. **(Q)** Representative images of intracellular ROS levels of chondrocytes. **(R)** Toluidine blue staining of ECM in chondrocytes. Statistical significance was calculated using two-tailed t-test, or one-way ANOVA with Tukey’s multiple comparisons test: * p < 0.05, *** p < 0.001. N=3, n=3.

As local injection of naked siRNA is susceptible to nuclease degradation and poor *in vivo* delivery efficiency. To address this, si-*SIRT3*@SPIONs were designed and fabricated. In brief, SPIONs were first modified with APTES to introduce amine groups, followed by activation with glutaraldehyde. Meanwhile, si-*SIRT3* was conjugated to the functionalized SPIONs via covalent bonding, and eventually synthesized si-*SIRT3*@SPION nanocomplex **(Figure 6A)**. With this synthesis approach, the encapsulation efficiency (EE%) and loading capacity (LC%) of si-*SIRT3*@SPIONs are 61.45% **(Figure S7A)** and 9.48% **(Figure S7B)**, respectively. Transmission electron microscopy (TEM) revealed that si-*SIRT3*@SPIONs exhibited distinct morphological changes compared to bare SPIONs, attributable to the successful conjugation of si-*SIRT3* **(Figure 6B)**. Both SPIONs and si-*SIRT3*@SPIONs demonstrated strong paramagnetic behavior under an external static magnetic field, supporting their potential for magnetic targeting under EMF exposure **(Figure 6C)**. Elemental mapping analysis confirmed that si-*SIRT3*@SPIONs contained not only Fe and O (primary components of Fe_3_O_4_), but also Si and N, indicating successful SiO_2_ coating and siRNA conjugation **(Figure 6D, E)**. This finding was further supported by co-localization analysis **(Figure 6F, G)**. Particle size distribution analysis showed a slight increase in the diameter of si-*SIRT3*@SPIONs (*approx.* 53.1 nm) compared to unmodified SPIONs (*approx.* 45.9 nm), likely due to the functionalized SiO_2_ layer and bound siRNA **(Figure 6H)**. Zeta potential analysis indicated a higher absolute value for si-*SIRT3*@SPIONs, suggesting improved stability and reduced aggregation tendency **(Figure 6I)**. Magnetic hysteresis curves confirmed the retained superparamagnetic properties of si-*SIRT3*@SPIONs **(Figure 6J)**. Moreover, X-ray diffraction (XRD) patterns exhibited characteristic diffraction peaks confirming the crystalline structure of the nanoparticles and successful functionalization **(Figure 6K)**. Fourier transform infrared (FT-IR) spectroscopy revealed the presence of key vibrational bands corresponding to P-O, P=O, and C=O bonds, further verifying the conjugation of siRNA to the functionalized SiO_2_-SPION complex** (Figure 6L)**. Moreover, the fluorescent images and co-locolization analysis of FAM-labeled siRNA@SPIONs further verified the synthesis method **(Figure S8)**. In addition, the stability of si-*SIRT3*@SPIONs in a physiological microenvironment was evaluated. There was no significant decrease in the fluorescent intensity of FAM-labeled siRNA@SPIONs over 48 h in the culture medium containing 10% FBS, confirming an *in vivo* stability **(Figure S9A)**. We also evaluated the protective effects of this nanocomplex against ribonuclease (RNase) degradation. The results indicated that the RNase degradation was not significant until 48 h **(Figure S9B)**. The comparable cell viability in different time points further support the stability of si-SIRT3@SPIONs **(Figure S9C)**.

To evaluate the magnetofection efficacy, we conducted a static magnetic field (SMF) induced magnetofection. Although the results demonstrated that chondrocytes have internalized SPIONs, but the SPIONs intend to agglomerate under SMF exposure **(Figure S10)**. To prevent SPION aggregation and improve its efficacy, the magnetofection of EMF with different parameters (*i.e.*, intensity and frequency) was screened and compared with traditional transfection. And the results indicated that 40 Hz and 50 Gauss EMF exhibited satisfactory magnetofection efficacy, and were used for subsequent research **(Figure S11)**. The *in vitro* therapeutic efficacy of si-*SIRT3*@SPIONs with or without EMF exposure was subsequently evaluated. Cell viability assays indicated good biocompatibility of si-*SIRT3*@SPIONs ± EMF treatment in chondrocytes **(Figure 6M)**. Live/dead staining showed a small number of dead cells in the control and EMF group, and there were no dead cells observed in the si-*SIRT3*@SPION ± EMF group, which are the normal levels of apoptosis **(Figure 6N)**. Cytoskeleton staining revealed no significant structural damage or cytotoxicity across all treatment conditions** (Figure 6O)**. Notably, immunofluorescence staining demonstrated that EMF exposure alone did not affect SIRT3 protein expression, whereas si-*SIRT3*@SPIONs slightly reduced SIRT3 levels. This knockdown effect was further significantly enhanced under EMF exposure **(Figure 6P)**. Furthermore, ROS staining indicated that si-*SIRT3*@SPIONs, particularly in combination with EMF, reduced oxidative stress in chondrocytes **(Figure 6Q)**. Toluidine blue staining revealed enhanced extracellular matrix (ECM) synthesis in the si-*SIRT3*@SPION + EMF group, suggesting promoted chondrogenic activity **(Figure 6R)**. While si-NC@SPIONs was used to exclude the potential effects of EMF exposure and the magnetofection **(Figure S12)**, it further confirmed the specificity of si-SIRT3@SPIONs. In summary, we successfully fabricated si-*SIRT3*@SPIONs with enhanced stability and delivery efficiency. This bioengineered nanocomplex under EMF exposure exhibited excellent biocompatibility and significantly improved siRNA delivery performance *in vitro*.

### T-2 toxin disrupts skeletal development in a KBD model

**Figure 7:**
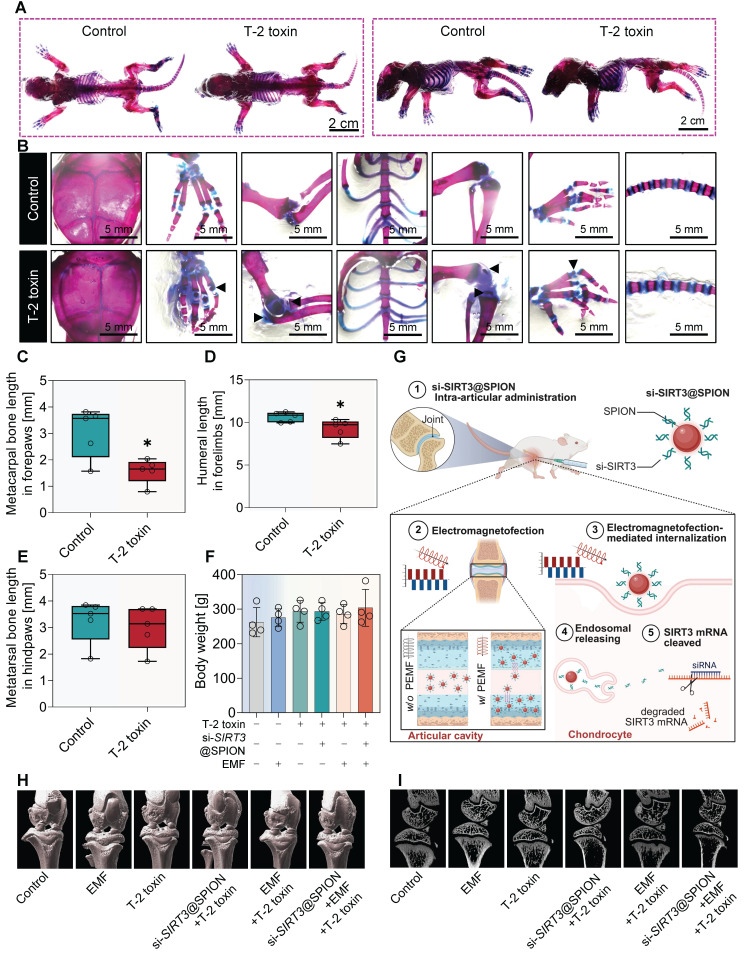
***In vivo* evaluation of the influences of T-2 toxin on SD rat skeletal development. (A)** Representative whole-mount skeletal staining images of 10-day-old SD rats using Alcian Blue (cartilage) and Alizarin Red (mineralized bone). **(B)** High-magnification views of local skeletal sites showing cartilage and bone structures. Quantitative analysis of the lengths of the **(C)** metacarpal bone, **(D)** humeral, and **(E)** metatarsal bone in control and T-2 toxin-treated SD rats, compared with the control. **(F)** Body weight of SD rats after the treatment of si-*SIRT3*@SPION with/without EMF. **(G)** *In vivo* experimental principle and workflow. **(H)** Three-dimensional CT reconstruction of knee joint. **(I)** Parasagittal micro-CT sections of knee joints from SD rats after treatment with si-*SIRT3*@SPIONs with/without EMF. Statistical significance was calculated using two-tailed t-test, * p < 0.05. N=3, n=3.

The establishment of a reliable animal model for KBD is crucial for advancing the in-depth research. To reach this goal, we performed whole-mount skeletal staining with Alcian Blue and Alizarin Red on 10-day-old SD rats **(Figure 7A)**. Local tissue examination revealed no significant difference in body weight, delay in fontanelle closure, and no notable morphological differences in the thoracic, caudal vertebrae, femur, forepaws, and hindpaws between T-2 toxin and control groups **(Figure S13)**. Interestingly, distinct ossification centers were observed in the metacarpophalangeal and metatarsophalangeal joints, as well as in the elbow and knee joints, of rats in the T-2 toxin group. In contrast, fusion of these ossification centers was already evident in the control group **(Figure 7B)**. Furthermore, the lengths of the metacarpal bone **(Figure 7C)** and humerus** (Figure 7D)** were shorter in the T-2 toxin group compared to the controls, although no difference was detected in metatarsal bone length **(Figure 7E)**. Collectively, these skeletal developmental features indicate that neonatal rats of T-2 toxin group exhibit clear signs of developmental delay.

Subsequently, the therapeutic efficacy of si-*SIRT3*@SPIONs was evaluated *in vivo*. The body weight of SD rats remained similar in each group, and the EMF exposure alone had no significant effects **(Figure 7F)**. The *in vivo* experimental principle and workflow is performed as the schematic illustration showed, pregnant SD rats were fed with or without T-2 toxin, the offspring were maintained on the same diet post-weaning. At their 4 weeks of age, T-2 toxin-dieted SD rats received intra-articular injections of si-*SIRT3*@SPIONs and/or EMF exposure. Under EMF exposure, si-*SIRT3*@SPIONs can be remotely controlled to locally deliver si-*SIRT3* into the cartilage for precisely protecting chondrocytes **(Figure 7G)**. As the knee joint is one of the most frequently affected sites in KBD patients, we first evaluated the osseous condition of the knee joint with three-dimensional CT reconstruction **(Figure 7H)** and images of parasagittal CT sections **(Figure 7I)**. However, the EMF exposure alone has no significant effects. Moreover, no significant change in the structure or quantitative data **(Figure S14)** was detected in the T-2 toxin group, which may be attributed to the early disease stage of the animal model. More pronounced bone destruction might manifest at later stages or in aging models.

### si-*SIRT3*@SPION combined with EMF exposure ameliorates cartilage damage *in vivo*

**Figure 8:**
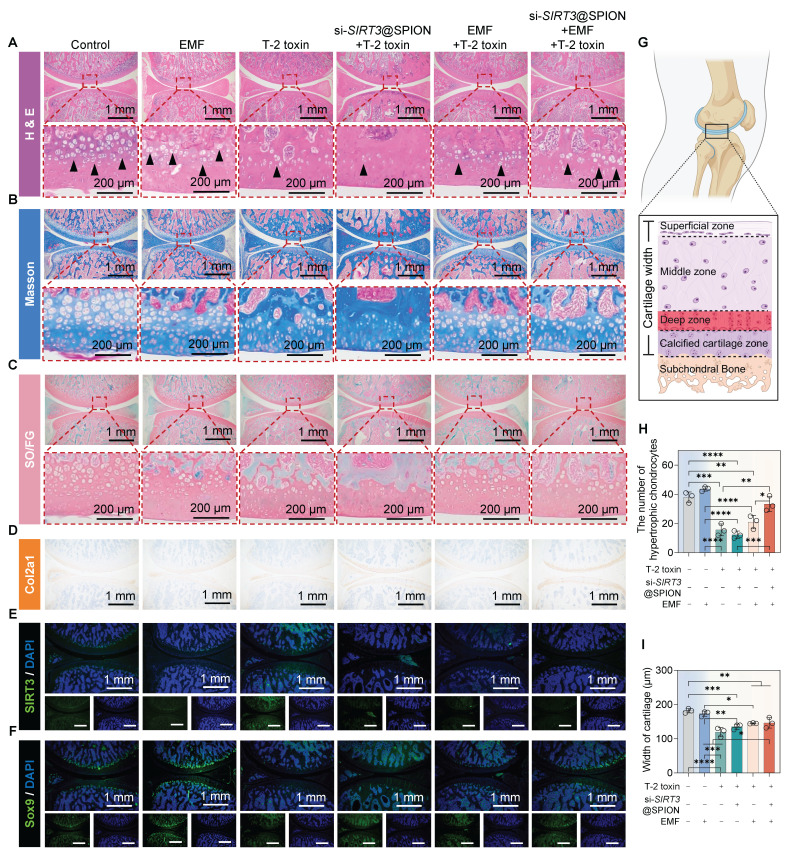
***In vivo* evaluation of the protective effects of si-*SIRT3*@SPION and EMF exposure on knee joint cartilage. (A)** Representative H&E staining of the knee joint. **(B)** Representative Masson trichrome-stained sections of the knee joint. **(C)** Representative Safranin O Fast Green sections of proteoglycan distribution. **(D)** Immunohistochemical detection of Col2a1 expression in articular cartilage. **(E)** Immunofluorescence staining of SIRT3 in cartilage tissues. **(F)** Immunofluorescence staining of Sox9 in cartilage tissues. **(G)** Schematic diagram illustrating the structure of knee joint cartilage. **(H)** Quantitative analysis of cell number in the deep zone of the knee joint cartilage. **(I)** Quantitative measurement of cartilage thickness. Statistical significance was calculated using one-way ANOVA with Tukey’s multiple comparisons test: * p < 0.05, ** p < 0.01, *** p < 0.001. N=3, n=3.

To assess the therapeutic effects of si-*SIRT3*@SPION under EMF exposure in an animal model of KBD, the morphological features of knee joint cartilage from SD rats across experimental groups were examined. H&E staining revealed a smooth cartilage surface in all groups. However, a reduction in both the number and size of cartilage lacunae was observed in the T-2 toxin-treated group and the si-*SIRT3*@SPION + T-2 toxin group, whereas both the number and size of cartilage lacunae were markedly increased in the si-*SIRT3*@SPION + EMF + T-2 toxin group **(Figure 8A)**.

Masson trichrome staining **(Figure 8B)** and Safranin O Fast Green staining **(Figure 8C)** further demonstrated a slight decrease in cartilage thickness following T-2 toxin treatment, with noticeable recovery after treatment with si-*SIRT3*@SPION combined with EMF exposure. Immunohistochemical staining for Col2a1 confirmed the protective effect of si-*SIRT3*@SPION + EMF against T-2 toxin-induced extracellular matrix damage **(Figure 8D)**. Additionally, immunofluorescence staining of *SIRT3* revealed overexpression of *SIRT3* in cartilage tissue after T-2 toxin treatment, which was effectively restored by si-*SIRT3*@SPION + EMF treatment **(Figure 8E)**. Similarly, immunofluorescence for Sox9 indicated that si-*SIRT3*@SPION + EMF also significantly preserved Sox9 expression levels against T-2 toxin-induced damage **(Figure 8F)**.

Articular cartilage has different layers, including the superficial zone, middle zone, and deep zone, and calcified cartilage zone **(Figure 8G)**. The deep zone contains hypertrophic chondrocytes clusters adjacent to the subchondral bone. This area is critical for maintaining cartilage integrity and is particularly susceptible to damage under pathological conditions. Therefore, quantitative assessments of cartilage thickness and the number of chondrocytes in the deep zone were performed to evaluate the extent of cartilage damage and therapeutic efficacy. As the data showed, T-2 toxin significantly reduced the number of hypertrophic chondrocytes in the deep zone, the EMF exposure alone has no significant effects on cell number, but the damage caused by T-2 toxin was substantially reversed by si-*SIRT3*@SPION + EMF treatment **(Figure 8H)**. Consistent with these findings, cartilage thickness was also decreased by T-2 toxin and restored following si-*SIRT3*@SPION + EMF administration** (Figure 8I)**. To evaluate the systemic cytotoxicity of T-2 toxin and the treatment of si-*SIRT3*@SPION and EMF, the pathological examination by H&E staining of major organs was performed, the results showed no evidence of cytotoxicity** (Figure S15)**.

Together, these *in vivo* results demonstrate that T-2 toxin induces significant structural and compositional damage to articular cartilage, while the combination of si-*SIRT3*@SPION and EMF exposure exerts notable protective and therapeutic effects against the toxic effects caused by T-2 toxin.

## Discussion

Chondrocyte necrosis and apoptosis are considered as the main cause of KBD, with exposure of T-2 toxin, the levels of the apoptotic protein Fas, p53, and Bax increased, and the level of anti-apoptotic protein Bcl-xL decreased in chondrocytes 7. However, there still exists other mechanisms for the development of KBD. Transforming growth factor-β (TGF-β)/Smad signaling plays an important role in chondrogenic differentiation of MSCs and cartilage regeneration 36. TGF-β signaling is initiated through binding of ligands with TGF-βRI and TGF-βRII. It has been elucidated that T-2 toxin modulated TGF-β signaling to induce the abnormal chondrocyte hypertrophy and extracellular matrix degeneration 12. Our preliminary data indicated that T-2 toxin significantly impair TGF-β/Smads signaling pathway by up-regulating Smad7 **(Figure S16)**. In this study, transcriptome sequencing analysis identified *SIRT3* as the most significantly differentially expressed gene, suggesting a potential involvement of epigenetic mechanisms in the pathogenesis of KBD. SIRT3, a member of the class III HDACs, is an NAD^+^-dependent deacetylase that is ubiquitously expressed across various tissue cells. Its enzymatic activity is regulated by the cellular [NAD^+^]/[NADH] ratio 37. Although predominantly localized in mitochondria, SIRT3 has also been shown to bind and deacetylate substrates in the cytoplasm and nucleus 38. Previous studies have reported SIRT3 deficiency in chondrocytes may exacerbate the progression of OA, and it was proposed as a potential therapeutic target of maintaining homeostasis of cartilage 39. Nevertheless, the functional role of SIRT3 in modulating cell function and disease progress is multifaceted 40. For instance, *SIRT3* overexpression can interact with MDH2, resulting in the decrease of MDH2 acetylation and activity, thus leading to the disruption of the TCA cycle and mitochondrial dysfunction 41. Therefore, the overexpression of *SIRT3* impair the cellular metabolism, *e.g.,* increased ROS production 19, induction of ferroptosis 20, and ultimately cellular damage. To explore the upstream mechanism of *SIRT3* up-regulation by T-2 toxin, we performed histone methylation profiling and found no obvious influences on H3K27me3 or H3K4me3** (Figure S17)**. Furthermore, we assessed the gene expression related with m^6^A methylation modification and figured out Mettl3 as the differentially expressed gene **(Figure S18)**. It has been reported that SIRT3 can interact with METTL3, and affect the process of m^6^A methylation 42. Subsequently, we used deepSRAMP to figure out several potential sites of m^6^A modification in *SIRT3* mRNA **(Figure S19)**. Thus, the development of KBD may involve the m^6^A methylation modification, which is worthy of in-depth study in future. To further confirm the direct mechanism between METTL3-mediated m^6^A methylation and T-2 toxin-induced SIRT3 overexpression, we used si-METTL3 in chondrocytes. As the results showed, si-METTL3 significantly reduced METTL3 expression at both mRNA **(Figure S20A)**, and the protein levels of METTL3 and SIRT3 **(Figure S20B, C)**. Importantly, silencing METTL3 markedly reversed the overexpression of *METTL3* and *SIRT3* **(Figure S20D)**, and protein level induced by T-2 toxin **(Figure S20E-F)**. Pearson correlation analysis verified a positive correlation between METTL3 and SIRT3 expression **(Figure S20G)**. These results support our hypothesis that METTL3-mediated m^6^A modification enhances *SIRT3* mRNA stability and suppresses its degradation, thereby increasing *SIRT3* expression.

Based on the exploration in molecular mechanism, this study figured out the important role of SIRT3 in the development of KBD. Considering the side effects may be caused by pharmaceutical modulation of SIRT3, *e.g.*, lack of specificity and selectivity, and unfavorable pharmacokinetic properties. The RNA delivery is a promising approach to precisely modulate the specific gene in the specific tissue 43. However, the naked RNA is susceptible to rapid degradation by endogenous RNases and, due to its negatively charged nature, exhibits poor permeability across cell membranes 44. To address this challenge, Xiao *et al*. linked CXCR4-targeted p53 mRNA with NPs to achieve an improved biocompatibility and stability, to restore p53 function. This RNA-nanocomplex effectively activated CD8^+^ T cells, NK cells, and M1 macrophageto modulate the immune microenvironment 45. Islam *et al.* coated PTEN mRNA with a polymer-lipid hybrid NP to treat PTEN-null prostate cancer. This mRNA-NP complexes protected mRNA from RNase degradation and enable effective cellular uptake via macropinocytosis and endosomal escape, and eventually modulate PI3K-AKT signaling and cell apoptosis 46. Moreover, Tao *et al*. developed siRNA-NPs complexes for atherosclerotic therapy, this PLGA-lipid-PEG NPs protect si-*Camk2g* from nuclease degradation, prolong its circulation, specifically target macrophages, and effectively silent CaMKIIγ to modulate efferocytosis for advanced atherosclerosis 47.

In the joint cavity, the high viscosity of synovial fluid hinders the passive diffusion and distribution of NPs 48. Moreover, the cartilage ECM has a compact mesh pore size (~200 nm), which severely restricts the passive penetration of NPs with larger diameter, especially into the deep cartilage zone to protect the hypertrophic chondrocytes damaged by KBD 49. To overcome the diffusion barriers, EMF was applied because it can generate a directional magnetic driving force, and this strategy has been reported to facilitate delivering miRNA-magnetic nanoparticles (MNPs) (particle diameter: 10.31 nm) into BMSCs and HUVECs for intervertebral fusion 50. In our study, we also used an external EMF to produce a directional magnetic driving forces to actively transport si-SIRT3@SPIONs through the synovial fluid. Importantly, si-SIRT3@SPIONs have a diameter around 53.1 nm, which is smaller than the pore size of cartilage ECM, and this particle size is capable to affect hypertrophic chondrocytes in the deep zone of cartilage 51. Moreover, the exposure to EMF can safely and transiently enhance cell membrane permeability, thereby promoting rapid cellular internalization of nanoparticles 52. Meanwhile, the use of SPIONs as magnetic carriers enables efficient delivery of exogenous genetic material into target cells, circumventing the limitations associated with viral vectors, *i.e.*, immunogenicity and potential toxicity 50. Moreover, the electromagnetized NPs exposed to 100 Hz EMF has been reported to activate histone acetyltransferase (HAT) 53. Taking these merits into consideration, the present study designed and fabricated si-*SIRT3*@SPION to improve the stability of si-*SIRT3* and promote its efficient delivery into chondrocytes under EMF stimulation.

To better simulate the native living environment and cellular conditions of KBD patients, we carefully designed an animal model. Specifically, pregnant SD rats were maintained on a selenium-deficient diet (with or without T-2 toxin) throughout gestation and continuing after delivery. The pups were nursed by the dams until weaning, after which the offspring were also fed the same diet as their dams. Interestingly, the animal model results not only demonstrated developmental retardation at specific skeletal sites in KBD rats but also revealed significant cartilage degeneration. In the 10-week-old KBD model, T-2 toxin exhibited no influences on the body weight of SD rats **(Figure S21)** and several major organs **(Figure S22)**. However, the images of micro-CT indicated that long-term exposure to T-2 toxin may lead to the destruction of bone microstructure **(Figure S23)**. With the establishment of KBD animal model, we evaluated the therapeutic potential of si-*SIRT3*@SPION combined with EMF exposure for KBD *in vivo*. The treatment effectively preserved cartilage integrity and function, demonstrating significant therapeutic effects.

In addition to treat and control the development of KBD, importantly, this magnetofection-mediated siRNA delivery strategy holds great potential for other endemic diseases with tissue-specific toxicity. For instance, the skeletal fluorosis, caused by fluoride-contaminated water and food is also an endemic health concern 54. The fluoride can compete with Mg^2+^ thus widely affecting enzymes associated with apoptosis (caspases, Bcl-2, p53), epigenetic regulators (DNMTs, HDACs) 55. Keshan disease, an endemic disease in northeast China, is a fatal dilated cardiomyopathy 56. There are 3 potent pathogenic mechanisms of Keshan disease, such as selenium deficiency, virus infection, or mycotoxin infection 57. In the molecular level, the dysfunction of selenoproteins (GPx and thioredoxin reductase) can lead to the oxidant stress, lipid peroxidation, and eventually myocardial mitochondrial damage 58. As a therapeutic strategy, EMF-enhanced NP approach provides a non-invasive, spatiotemporally controlled gene modulation specific tissues, overcoming anatomical barriers, such as the dense extracellular matrix and avascular structures. Beyond KBD, given the established ability of EMF to easily penetrate through skin and soft tissues, this magnetofection strategy can also be extended to treat other disease, such as skeletal fluorosis and Keshan disease. Although further validation in other diseases and optimization of targeting specificity are required, this study established an EMF-enhanced nanotherapeutic paradigm that may provide a potential therapeutic paradigm in this field.

In summary, this study identified *SIRT3* as a critically differentially expressed gene involved in KBD pathogenesis. We also developed a novel nanotherapeutic strategy, si-*SIRT3*-loaded SPIONs delivered under EMF exposure, which markedly attenuated cartilage damage in a newly designed KBD animal model. These findings not only deepen the mechanistic understanding of KBD but also offer a targeted, effective strategy for its treatment, potentially improving clinical outcomes for patients in endemic regions.

## Conclusions

T-2 toxin disrupts chondrocyte protein acetylation and primary cilia integrity, thereby promoting cartilage damage in KBD. In this study, EMF-augmented si-*SIRT3*@SPIONs enhance delivery stability and transfection efficiency. *In vivo*, this strategy protected the primary cilia integrity, maintains chondrocyte phenotype, and alleviates KBD-related cartilage degeneration. These findings clarify a key pathological mechanism of KBD and provide a safe, non-invasive, targeted therapeutic approach for KBD.

## Materials and Methods

### Cell culture

Primary chondrocytes were isolated from the knee joints of 1-day-old C57 mice. Briefly, neonatal mice were euthanized and sterilized superficially. The skin and muscle tissues were carefully dissected away to expose the knee joints, which were then excised. Articular cartilage was harvested from the joint surfaces and minced into fine fragments. The tissue fragments were first digested with 0.25% trypsin (Cat No. 25200056, Gibco, USA) for 30 min at 37 °C. Following trypsinization, the cartilage was further digested with Collagenase NB 4G (Cat No. S1746502, Nordmark, Germany) for 12 h under the same temperature conditions. The resulting cell suspension was filtered through a 200-mesh sieve and centrifuged at 500 × g for 5 min at room temperature. The pellet was resuspended and cultured in DMEM/F12 medium with 10% FBS at 37 °C in a humidified incubator with 5% CO_2_. The culture medium was refreshed twice weekly. Cells were passaged upon reaching 80–90% confluence, and chondrocytes at passages 3–5 were utilized for all follow-up assays.

### Cell viability

Cell viability was assessed using the Cell Counting Kit-8 (CCK-8; Cat No. C0038, Beyotime Biotechnology, China) according to the manufacturer’s instructions. Briefly, primary chondrocytes were seeded in 96-well plates and exposed to various concentrations of T-2 toxin (0, 12.5, 50, and 100 μg/L) for 24 h. Following treatment, the cells were gently washed three times with phosphate-buffered saline (PBS). Subsequently, CCK-8 working solution was added to each well, and the plates were incubated at 37 °C for 1 hour. Absorbance was measured at 450 nm using a microplate reader (PerkinElmer, USA).

### Calcein-AM/PI staining

The calcein-AM/PI staining was performed for primary chondrocytes (± T-2 toxin). In detail, cells were firstly washed 3 times with sterile PBS, then cells were stained by Calcein-AM/PI working solution (Cat No. L3224, Thermo Fisher Scientific, Shanghai, China) for 1 h at 37 °C in the dark. Afterwards, the fluorescent images were taken and documented with a fluorescence microscope (CLSM, Nikon, Japan).

### Intracellular Ca^2+^ staining

Chondrocytes were loaded with 2 µM Fluo-4 AM (Cat No. S1060, Beyotime Biotechnology, China) at 37 °C for 2 h. Then, the chondrocytes were gently washed 3 times with PBS and incubated in Hanks Balanced Salt Solution (with Ca^2+^, Cat No. C0219, Beyotime Biotechnology, China). The images of intracellular Ca*2+* staining were recorded by the fluorescent microscope (CLSM; Nikon, Japan).

### Cytoskeleton staining

Cytoskeleton staining was conducted for the evaluation of cellular structure. In detail, primary chondrocytes were gently rinsed three times with PBS and then incubated with 4% paraformaldehyde (PFA). Subsequently, cells were permeabilized by using Triton X-100 and 2% PFA. Afterward, chondrocytes were washed and incubated in 0.5 µg/mL Phalloidin (Cat No. Y249127-10μg, Beyotime Biotechnology, China) and DAPI (Cat No. C1006-10mL, Beyotime Biotechnology, China) for 1 hour at 37 °C in the dark. The image of cytoskeleton staining was acquired.

### Immunofluorescence staining

Chondrocytes were gently washed three times with PBS and fixed in 4% PFA for 10 min at room temperature. Following fixation, cells were permeabilized with 0.2% Triton X-100 for 10 min and refixed with 2% PFA for another 10 min. Non-specific binding sites were blocked by incubation with 5% bovine serum albumin (BSA) on a shaker for at least 1 hour at room temperature. Cells were then incubated overnight at 4 °C with the following primary antibodies: anti-Collagen II (1:100, rabbit, Cat No. 28459-1-AP, Proteintech, China), anti-Sox9 (1:500, rabbit, Cat No. ET1611-56, Huabio, China), and anti-SIRT3 (1:200, rabbit, Cat No. 10099-1-AP, Proteintech, China). After 1^st^ antibody incubation, cells were then incubated with a CoraLite 594-conjugated goat anti-rabbit secondary antibody (1:100, Cat No. SA00013-4, Proteintech, China) for 2 h in the dark. DAPI (5 µg/mL, Cat No. C1002, Beyotime Biotechnology, China) was used for nuclear counterstaining for 30 min in the dark. Immunofluorescence images were recorded with a fluorescence microscope.

For quantitative analysis, the immunofluorescence images were measuring the mean fluorescence intensity of individual cells. For each experimental group, 3 independent biological replicates (N = 3) were analyzed. In each replicate, 3 randomly selected non-overlapping image fields (N = 3) were chosen, and ≥ 30 cells per field were randomly selected and outlined, and the mean fluorescence intensity was measured using ImageJ software.

### Staining and analysis of primary cilia

To induce primary cilia formation, chondrocytes were serum-starved for 48 h before treatment. Chondrocytes were fixed with and permeabilized, then the non-specific binding was blocked by 5% BSA for at least 1 hour. Subsequently, cells were incubated overnight at 4 °C with an anti-Acetylated Tubulin primary antibody (1:200, mouse; Cat No. 66200-1-Ig, Proteintech, China). After washing with PBS, samples were incubated for 2 h in the dark with a CoraLite 488-conjugated goat anti-mouse secondary antibody (1:100; Cat No. SA00013-1, Proteintech, China), followed by 30 min nuclear staining of DAPI (5 μg/mL; Cat No. C1002, Beyotime Biotechnology, China). Immunofluorescence images were acquired with the fluorescence microscope. Primary cilia length and the proportion of ciliated cells were further measured with ImageJ software.

### DCFH-DA assay

Intracellular ROS levels in chondrocytes were measured using a fluorometric ROS assay kit (Cat No. E-BC-K138-F, Elabscience, China). Briefly, cells were gently washed three times with PBS and incubated with 10 μM 2′,7′-dichlorodihydrofluorescein diacetate (DCFH-DA) for 30 min at 37 °C in the dark. After incubation, cells were washed three times with PBS to remove excess probe. Fluorescence images were acquired using a fluorescent microscope (CLSM; Nikon, Japan).

### Toluidine blue staining

Following a gentle wash with PBS, chondrocytes were fixed in 4% PFA then rinsed thoroughly with ddH_2_O. Chondrocytes were then incublated with a toluidine blue solution (Cat. No. C0637-100mL, Beyotime Biotechnology, China). After an incubation for 30 min, samples were rinsed with ddH_2_O to remove residual dye. Bright-field images were recorded using an inverted light microscope.

### RNA isolation and RT-qPCR

Total RNA was extracted from chondrocytes using the Eastep® Super Total RNA Extraction Kit (Cat No.LS1040, Promega Biotech, Beijing, China) following the manufacturer’s protocol. RNA concentration and purity were determined using a NanoDrop™ spectrophotometer (Thermo Fisher Scientific, China). cDNA was harvested from the reverse-transcribed RNA, by using the First Strand cDNA Synthesis Kit (Cat No. D7190S, Beyotime Biotechnology, China). Then, the SYBR Green (Cat No. D7268S, Beyotime Biotechnology, China) was used to perform qRT-PCR. The 2^-ΔΔCT^ method was used to calculate the relative gene expression, with GAPDH serving as the internal control. All primer sequences are summarized in Table 1.

### Western blot

Chondrocytes were lysed using RIPA buffer (Cat No. PC101, Epizyme Biotech, Shanghai, China) supplemented with protease and phosphatase inhibitor cocktails (Cat No. GRF103, Epizyme Biotech, Shanghai, China). The lysate was centrifuged at 3000×g for 10 min, and the protein concentration of the supernatant was determined using a BCA protein assay kit (Cat No. ZJ102, Epizyme Biotech, Shanghai, China). Subsequently, 25 µg of total protein from each sample was separated by SDS-PAGE (10% or 15% acrylamide-bisacrylamide gels) at 120 V for 150 min. The separated proteins were then transferred onto 0.22 μm PVDF membranes at 400 mA for 30 min using an ice-bath free Western Superquick Transfer Buffer (Cat No. PS201S, Epizyme Biotech, Shanghai, China). Subsequently, the unspecific binding sites on PVDF membrane were blocked with 5% BSA on a shaker at room temperature for at least 2 h. The detailed information about antibodies that used in this study is summarized in Table 2.

### Fabrication and calibration of EMF exposure system

The custom-designed EMF exposure system comprised 4 main components: a one-dimensional Helmholtz coil (custom model based on Cat. No. DXHC15-10, Dexin Mag, Xiamen, China), a solenoid (custom design, Dexin Mag, Xiamen, China), a power amplifier (Cat. No. DXACP-0332, Dexin Mag, Xiamen, China), and a multi-waveform generator (Cat. No. 8300S, Dexin Mag, Xiamen, China). The magnetic field intensity was calibrated prior to each experiment using a Gaussmeter (Cat. No. DX-102F, Dexin Mag, Xiamen, China).

### Transcriptome sequencing and analysis

Total RNA was isolated with TRIzol Reagent (Cat. No. R0016, Beyotime Biotechnology, China), and its quality was verified using an Agilent 5300 Bioanalyzer and NanoDrop ND-2000. Only high-quality RNA samples (OD260/280 = 1.8–2.2, OD260/230 ≥ 2.0, RQN ≥ 6.5, 28S:18S ≥ 1.0, total RNA > 1 μg) were used for library construction. Sequencing libraries were prepared using either the Illumina® Stranded mRNA Prep Ligation Kit (1 μg input) or the SMART-Seq V4 Ultra Low Input RNA Kit (10 ng input), following the manufacturers protocols. Libraries were firstly quantified with Qubit 4.0, then were sequenced with the Illumina NovaSeq X Plus platform in the paired-end 150 bp mode. Raw reads were quality-trimmed using fastp, and clean reads were aligned to the reference genome using HISAT2. Transcript assembly and expression quantification were performed with StringTie and RSEM, respectively. The analysis of differential gene expression (DEGs) was conducted by DESeq2 and DEGseq. The significant DEG was defined as |log2FC| ≥ 1 and FDR ≤ 0.05 (DESeq2) or FDR ≤ 0.001 (DEGseq). Lastly, GO and KEGG analysis were performed using DAVID Bioinformatics.

### Synthesis and characterization of si-*SIRT3*@SPIONs

Firstly, 10 mg/mL SPION (Cat. No. 725358, Merck, Germany) were washed 3 times with ethanol and ddH_2_O. SPIONs were then dispersed in 10 mL of ethanol, followed by the addition of 100 µL of APTES (Cat. No. 741442, Merck, Germany). The mixture was stirred at room temperature for 6 h. The APTES-modified SPIONs were magnetically separated, washed with ethanol, and redispersed in PBS (0.1 M, pH 7.4). 1 mg of Aminocaproic acid (Cat. No. HY-B0236, MCE, China) was dissolved in 100 µL MES buffer (Cat. No. ST370-100mL, Beyotime, China) was then activated using 1.5 mg EDC (Cat. No. ST1299-5g, Beyotime, China) and 0.9 mg NHS (Cat. No. 130672, Merck, Germany) for 30 min. Then, 10 nmol of siRNA (in 50 µL DEPC water, Cat. No. ST036, Beyotime, China) was added and reacted for 2 h. The samples were precipitated with sodium acetate (3 M, pH 5.2, Cat. No. S2889, Merck, Germany) and cold ethanol at -20 °C overnight. Afterwards, samples were centrifuged, washed with 70% ethanol, and dissolved in DEPC water. Subsequently, APTES-modified SPIONs (1 mL, 10 mg/mL) were activated with 10 µL of glutaraldehyde (25%) for 1 hour. After washing, the particles were incubated with 50 µL of aminated siRNA (10 nmol) in PBS for 4 h. The conjugate was magnetically separated, washed with PBS, and resuspended in 1 mL PBS.

The morphology and elemental distribution of the nanoparticles were characterized by transmission electron microscopy (TEM, JEOL JEM-F200, Japan). Elemental co-localization was quantified using ImageJ software. The particle sized distribution and zeta potential were measured with a dynamic and electrophoretic light scattering analyzer (Malvern Zetasizer Nano ZS90, UK). The magnetic hysteresis curves were evaluated with a vibrating sample magnetometer (LakeShore 8604, USA). The chemical composition, as well as the elemental analysis, were characterized using an X-ray diffractometer (XRD, Rigaku SmartLab SE, Japan) and a fourier transform infrared spectroscopy (Thermo Fisher Scientific Nicolet iS20, USA).

### Encapsulation efficiency and loading capacity

The encapsulation efficiency (EE%) and loading capacity (LC%) of si-*SIRT3*@SPION were determined using a magnetic separation method. In brief, 1 mL of the si-SIRT3@SPION suspension in a 1.5 mL EP tube. After a complete magnetic separation of si-SIRT3@SPION, the supernatant containing unencapsulated siRNA was carefully collected. The separated pellet was then carefully washed twice with 1 mL PBS, and the washing solutions were mixed with the previous supernatant to completely collected all unencapsulated siRNA. The combined solution was centrifuged at 12000 × g for 10 min at room temperature to remove potential SPION, and the resulting clear supernatant was used for free siRNA quantification. To measure the total siRNA in si-SIRT3@SPION,1 mL of the si-SIRT3@SPION suspension was lysed with 1% SDS for 30 min with shaking. The lysate was subsequently centrifuged (12000 × g, 10 min), then the supernatant was carefully collected for total siRNA quantification. The siRNA concentration in the free and total supernatant samples, and the background were measured using a NanoDrop. The amount of encapsulated siRNA was calculated by subtracting the free siRNA amount from the total siRNA amount. The EE% and LC% were then calculated using the following equations:



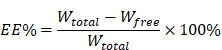





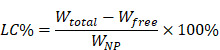



W_total_ is the total siRNA amount initially added and lysed by SDS, W_free_ is the free siRNA amount determined from the mixed supernatant, and W_NP_ is the total mass of SPION in the corresponding aliquot.

### Evaluation of nanocomplex stability *in vitro*

To evaluate the stability of the si-*SIRT3*@SPION nanocomplex under physiological conditions, the stability assays were performed. FAM-labeled siRNA@SPIONs were added in DMEM/F-12 culture medium supplemented with 10% fetal bovine serum (FBS, Cat No. 16010159, Gibco, USA) and incubated at 37 °C in a humidified incubator with 5% CO_2_ in the dark. At different time points (0, 12, 24, and 48 h) after cell attachment, the fluorescence images of FAM-labeled siRNA@SPIONs were captured using a fluorescence microscope (CLSM, Nikon, Japan), and quantified using ImageJ software. In addition, the protective effect of the nanocomplex against intracellular ribonuclease (RNase) degradation was evaluated. Primary chondrocytes were seeded in 24-well plates at a density of 4 ×10^4^ cells per well for 24 h, and then transfected with FAM-labeled siRNA@SPIONs under EMF-enhanced magnetofection as described above. After 24 h of incubation, cells were gently washed 3 times with PBS. The fluorescent images were taken at different time points, and the percentage of FAM-positive cells was quantified using ImageJ software.

### Human specimen

Knee joint tissues were obtained from 2 patients (1 with OA and 1 with KBD) who underwent total knee arthroplasty (TKA) due to severe joint deformity and loss of mobility. The diagnoses of OA and KBD were confirmed by 2 independent senior orthopedic surgeons and 2 radiologists. Following intraoperative osteotomy during TKA, bone and cartilage fragments from the distal femur and tibial plateau were collected and immediately preserved in formalin. All personally identifiable information was removed at the time of sample acquisition. All procedures involving human specimens were conducted in accordance with the guidelines of the Ethics Committee of Sichuan University (Approval No. 2022422) and conformed to the ethical principles of the 2008 Declaration of Helsinki.

### Animal experiment

The animal experiment in this study was modified based on the established KBD model 59. Pregnant Sprague-Dawley (SD) rats were purchased from Dashuo Laboratory Animal Co., Ltd. All animals were maintained under controlled conditions (temperature: 23–25 °C; humidity: 40-60%; 12 h light/dark cycle) and supplemented with or without 0.1 mg/kg T-2 toxin. Throughout gestation, dams remained on their respective diets. After delivery, pups were nursed by their biological dams until weaning, after which offspring continued to receive the same diet as their dams.

To investigate the developmental toxicity of T-2 toxin on endochondral ossification during gestation and lactation. At the time of 10 days after delivery, the neonatal SD rats of control group and T-2 toxin group were sacrificed for the whole-mount skeletal staining. In detail, neonatal SD rats were skinned and eviscerated, with the skeletal and cartilaginous structures retained. The specimens were rinsed thoroughly with PBS and subsequently fixed. After fixation, the samples were stained in 0.1% Alcian blue solution at room temperature for 24 h. Excess dye was then removed by washing in a solution of 80% ethanol and 20% acetic acid until the background became transparent. Subsequently, the specimens were stained with 0.1% Alizarin red solution for 24 h at room temperature, followed by a brief rinse in 95% ethanol to eliminate residual dye. Samples were then immersed in 1% KOH for 3 days, with daily monitoring. If necessary, the KOH solution was replaced to achieve complete tissue clearing. The cleared specimens were then sequentially transferred through a graded glycerol series (20%, 50%, and 80%) and finally preserved in 100% glycerol. Imaging was performed using a stereomicroscope, and subsequent morphological analyses were conducted with ImageJ software.

At 4 weeks postpartum, the skeletal development of SD rat is close to matured, and the knee joint cavity is sufficiently large to perform intra-articular injections. The offspring from T-2 toxin-exposed dams were randomly divided into one of the following experimental groups: (1) T-2 toxin, (2) T-2 toxin + si-*SIRT3*@SPION, (3) T-2 toxin + EMF, (4) T-2 toxin + si-*SIRT3*@SPION + EMF.

For intra-articular delivery, 25 μL of si-*SIRT3*@SPION suspension in sterile PBS was administered into the right knee joint using an insulin syringe with a 29-gauge needle. The injection was performed twice weekly. For EMF exposure, SD rats were placed inside a Helmholtz coil and subjected to EMF stimulation for 30 min per day. The treatment was conducted for 3 weeks. Subsequently, other SD rats were maintained on their respective diets (with or without T-2 toxin) for a total of 10 weeks to facilitate the development of the late-stage disease model. The procedure of the animal experiment was carefully conducted in accordance with the guidelines of the Animal Ethics Committee of Sichuan University (Approval No. 20220309002).

### micro-CT evaluation

The bone tissue of the knee joint was assessed using Micro-CT (NMC-100, PINGSENG Healthcare, Shanghai, China). In brief, the parameters of micro-CT were set as 80 kV and a current of 0.08 mA. All samples were scanned using micro-CT with identical parameters applied to the appropriate regions of interest (ROI). Imaris (version: 10.2, Oxford Instruments, USA) software was used for the subsequent analysis of Micro-CT results.

### Histological analysis

All tissue specimens were immersed in 10% EDTA for 30 days to achieve decalcification. After which they were paraffin-embedded and sectioned into 10-µm-thick slices. Sections were subjected to H&E staining, Safranin O fast green staining, Masson trichrome staining, immunohistochemistry, and immunofluorescence staining according to established protocols from our previous study 60. Histopathological images were acquired using a fluorescent microscope. The number of chondrocytes in the deep zone and the width of the cartilage was analyzed with Image J.

### Statistical analysis

The number of biological (N) and technical (n) replicates for each experiment is indicated in the corresponding figure legends. Error bars represent the standard deviation (SD) of the mean. Statistical significance was determined using either an unpaired two-tailed Student’s t-test (for two-group comparisons) or one-way analysis of variance (ANOVA) followed by Tukey’s post-hoc test (for multi-group comparisons). Statistically significance was considered at p < 0.05. Statistical analyses were performed with the GraphPad Prism software V.8.0.1 (El Camino Real, USA).

## Supplementary Material

Supplementary figures.

## Figures and Tables

**Scheme 1:**
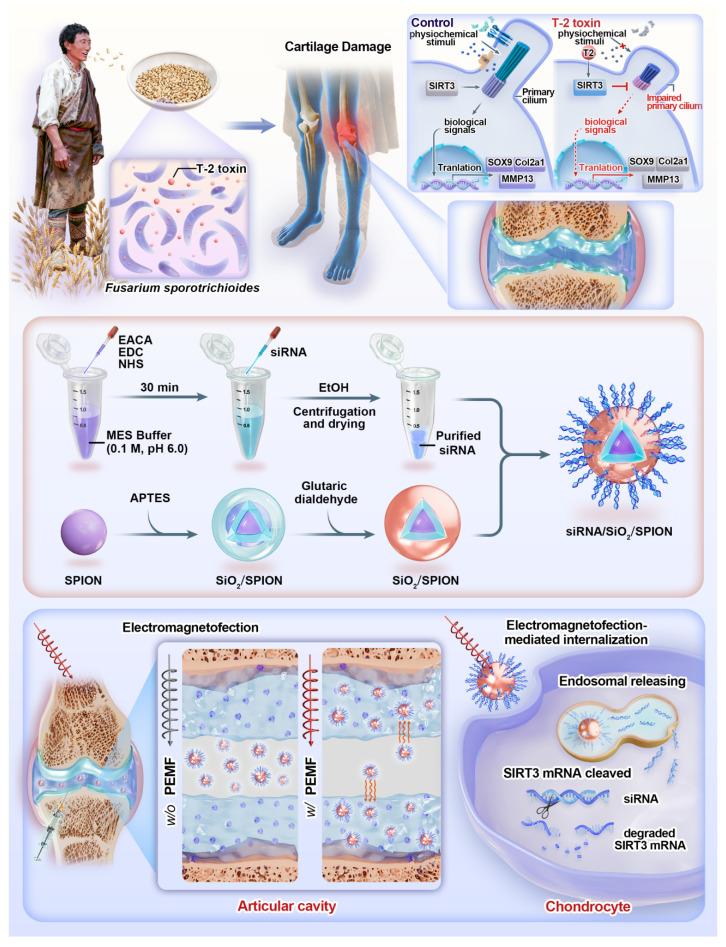
** Schematic diagram illustrating the synthesis and therapeutic principle for KBD.** In endemic regions, KBD patients dietary exposed to *Fusarium sporotrichioides*-contaminated grains leads to the ingestion of T-2 toxin. T-2 toxin is a mycotoxin which leads to the overexpression of *SIRT3* and damage of primary cilia in chondrocytes. Consequently, T-2 toxin results in the articular cartilage degeneration and loss of joint mobility in KBD patients. To overcome this challenge, the nanocomplex was fabricated by loading small interfering RNA targeting *SIRT3* (si-*SIRT3*) onto silica-coated superparamagnetic iron oxide nanoparticles (SPIONs). Upon intra-articular administration and application of an external EMF, the si-*SIRT3*@SPION complex was efficient transfected into chondrocytes of both upper and lower cartilage layers. This process enabled RNA interference-mediated silencing of *SIRT3*, which is pathologically overexpressed in the chondrocytes of KBD patients, thereby exerting a potential therapeutic effect.

**Table 1:** Primers used in RT-qPCR

Gene	Accession Number	Forward Primer (5’–3’)	Reverse Primer (5’–3’)
*GAPDH*	NM_017008.4	GCGAGATCCCGCTAACATCA	ATTCGAGAGAAGGGAGGGCT
*IFT20*	NM_001105815.1	TGTCGGCATGTAGTAGGCAC	GTGGTCTGCTGGGTAACCTC
*SIRT3*	NM_001106313.2	TGTGGGGTCCGGGAGTATTA	ATCACGTCAGCCCGTATGTC
*ARL13B*	NM_001107101.1	GCCTCTTGGTGAAACACGTCA	AGTCCACTGGCTCAACTTCTG
*MMP13*	NM_133530.1	TCCATCCCGAGACCTCATGT	CTCAAAGTGAACCGCAGCAC
*Col2a1*	NM_001414896.1	TGTGAAGACCCAGACTGCCT	TTCGCCACGAGAACCTTGAG
*Sox9*	NM_080403.3	ACAACGCAAGCTTCTGCAAG	GTGGGGCGAACAAACAAGAC
*METTL3*	NM_019721	CTGGGCACTTGGATTTAAGGAA	TGAGAGGTGGTGTAGCAACTT
si-*SIRT3*	/	CUUGUCUGAAUCGGUACAGAATT	UUCUGUACCGAUUCAGACAAGTT
si-*IFT20*	/	GAAUAUGAAGCUUUGUGUA	UACACAAAGCUUCAUAUUC
si-*METTL3*	/	GAGAUCCUAGAGCUAUUAA	UUAAUAGCUCUAGGAUCUC

**Table 2:** Antibody list

Antibody	Dilution	Source	Catalog No.
Acetyl-Tubulin	1:2000	mouse	66200-1-Ig (Proteintech, China)
IFT20	1:500	rabbit	13615-1-AP (Proteintech, China)
Collagen 2a1	1:2000	rabbit	28459-1-AP (Proteintech, China)
MMP 13	1:2000	rabbit	ET1702-14 (Huabio, China)
Sox9	1:10,000	rabbit	ET1611-56 (Huabio, China)
ARL13B	1:1000	rabbit	17711-1-AP (Proteintech, China)
SIRT3	1:1000	rabbit	10099-1-AP (Proteintech, China)
Tubulin	1:5000	rabbit	11224-1-AP (Proteintech, China)
GAPDH	1:50,000	mouse	60004-1-Ig (Proteintech, China)
Acetyl-Lysine	1:1000	rabbit	PTM-105RM (PTM BIO, China)
METTL3	1:1000	rabbit	ab195352 (Abcam, UK)
