# Supplementary material for: Magnetofection-mediated siRNA delivery ameliorates cartilage damage in Kashin-Beck disease via targeting SIRT3 and protecting primary cilia
Source: Theranostics. 2026 Jul 22;16(14):8302–25. doi: 10.7150/thno.134335 (PMC13440625; doi:10.7150/thno.134335)
Supplement: Supplementary file 1 [file thnov16p8302s1.pdf]

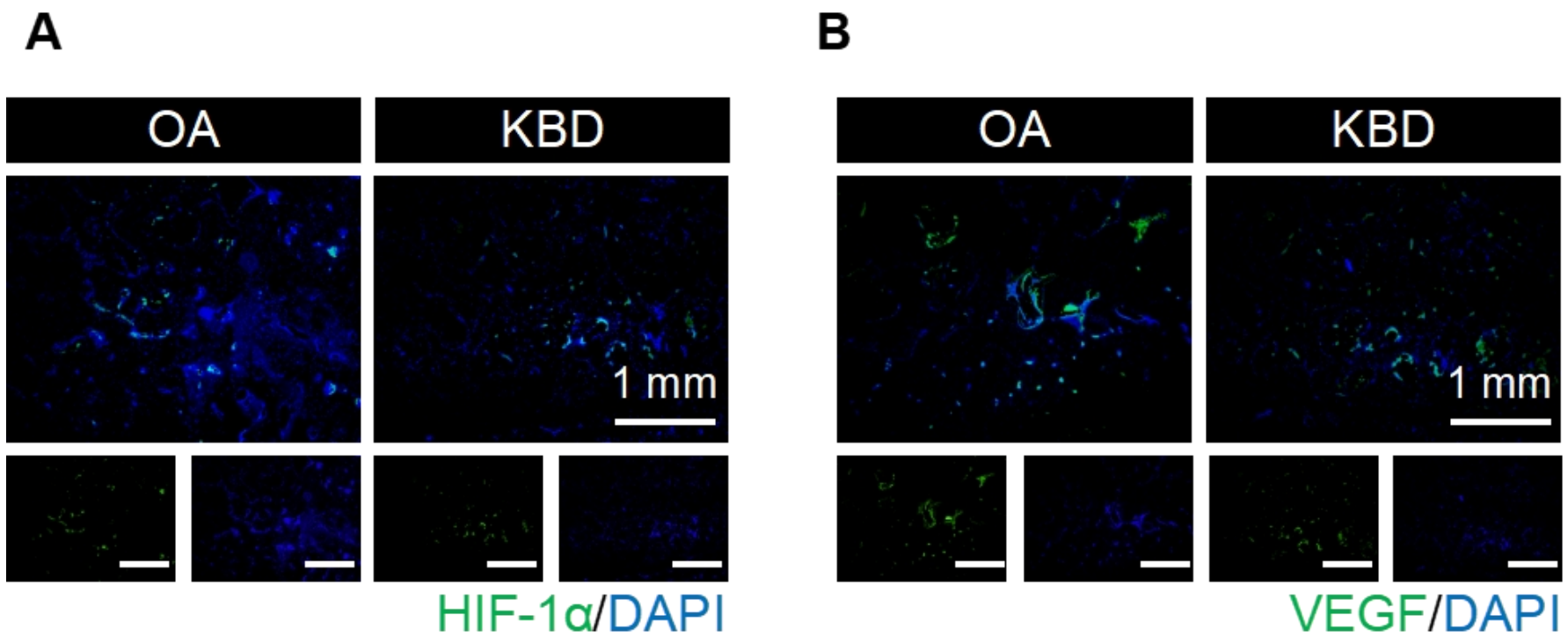

Figure S1. Immunofluorescent staining for **(A)** HIF-1α, and **(B)** VEGF in the femoral condyle cartilage in patients with KBD and OA.

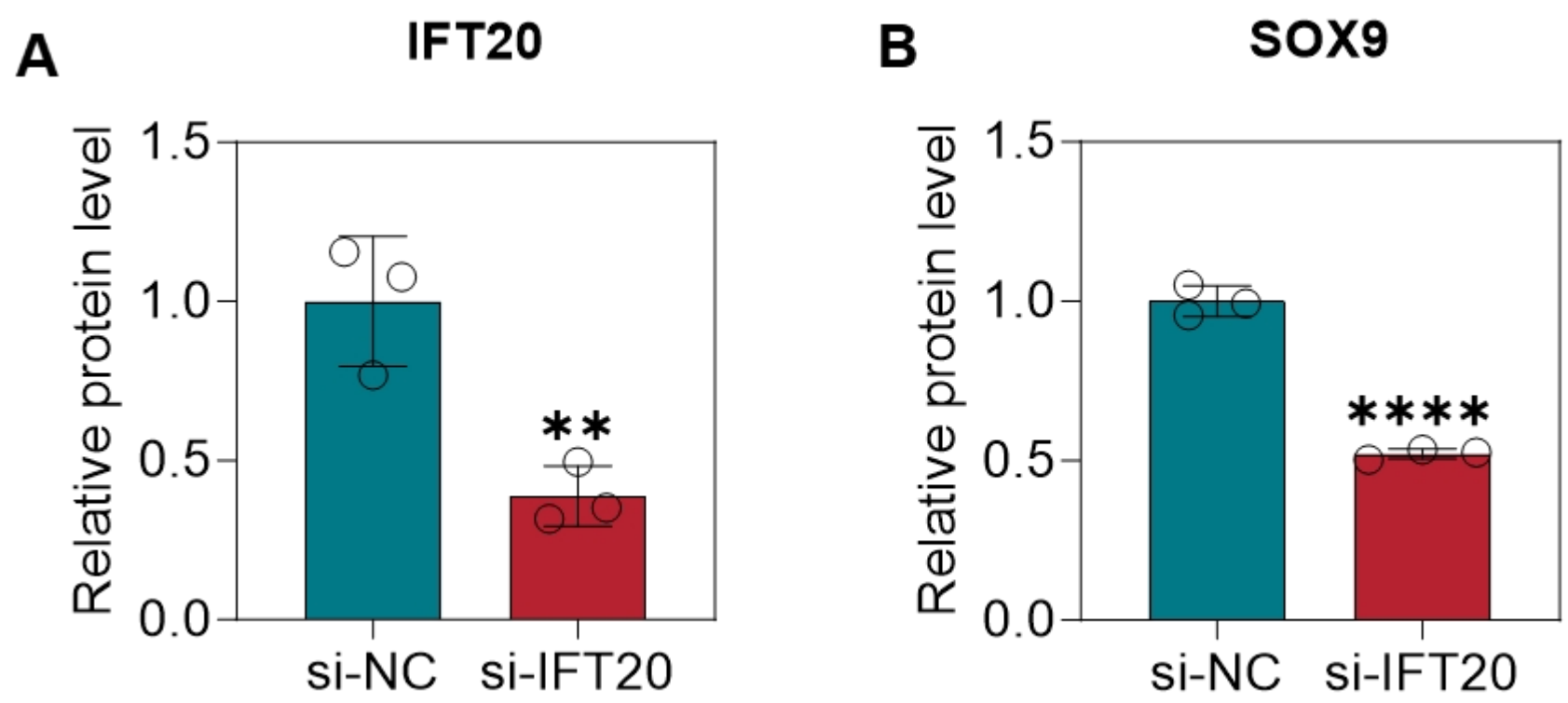

Figure S2. Quantitative data of **(A)** IFT20, and **(B)** SOX9 protein level in chondrocytes treated with si-NC or si-IFT20. $N \geq 3$, $n = 3$. Data were compared by unpaired, two-tailed t-test: ** $p < 0.01$, **** $p < 0.0001$.

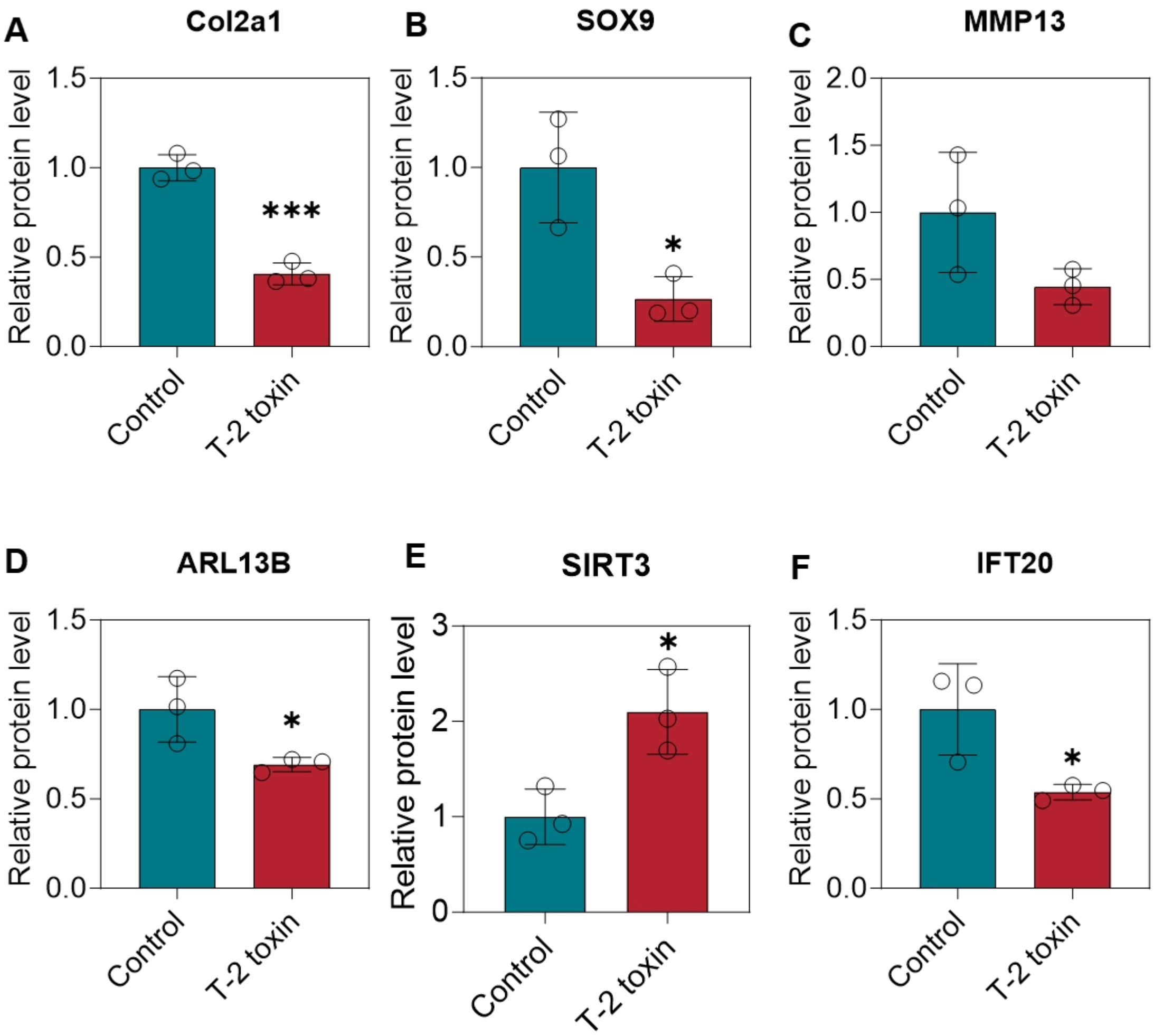

Figure S3. Quantitative data of **(A)** Col2a1, **(B)** SOX9, **(C)** MMP13, **(D)** ARL13B, **(E)** SIRT3, and **(F)** IFT20 protein level in chondrocytes treated with or without T-2 toxin. N ≥ 3, n = 3. Data were compared by unpaired, two-tailed t-test: * $p < 0.05$, ** $p < 0.001$.

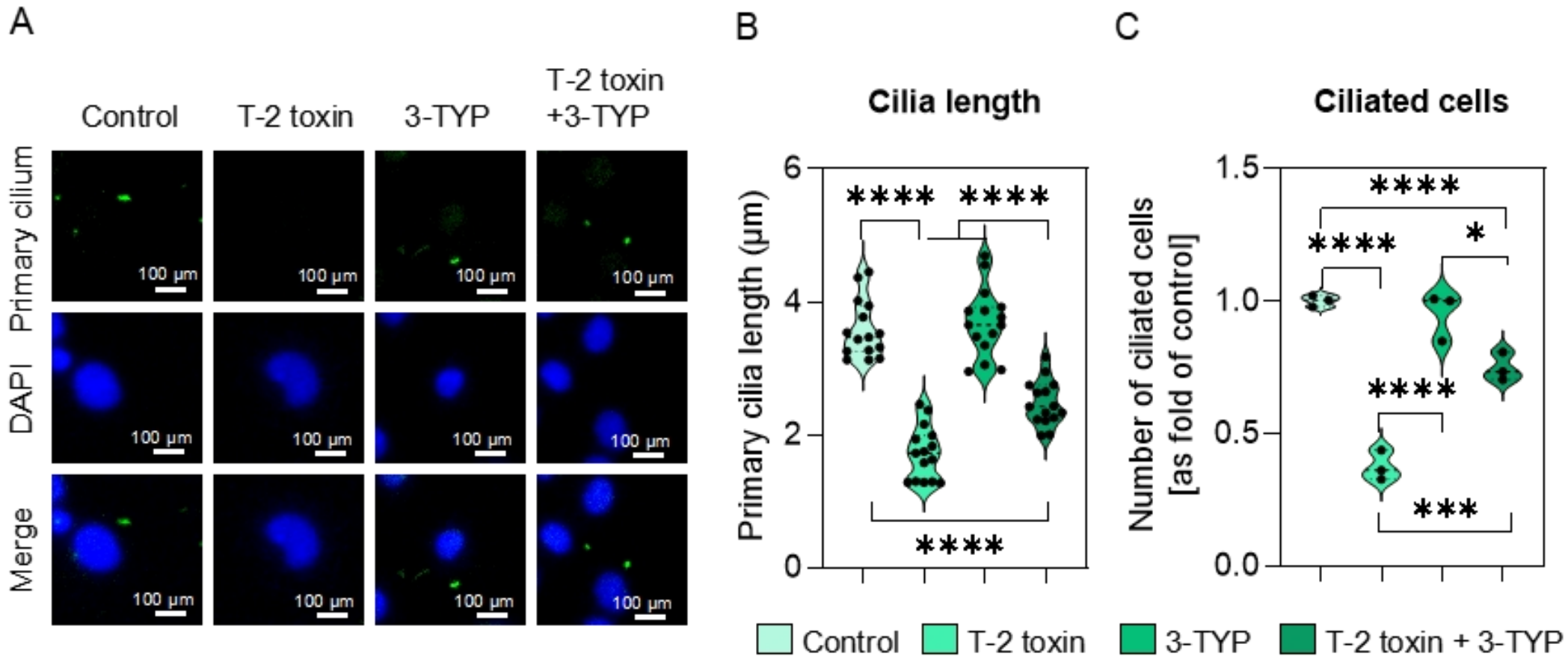

Figure S4. Immunofluorescence analysis of primary cilia **(A)** revealed that 3-TYP treatment attenuated T-2 toxin-induced ciliary damage, preserving both **(B)** cilium length and **(C)** the proportion of ciliated cells. N ≥ 3, n = 3. Data were compared by non-parametric one-way ANOVA followed by Tukey's multiple comparison test: * $p < 0.05$, *** $p < 0.001$, **** $p < 0.0001$.

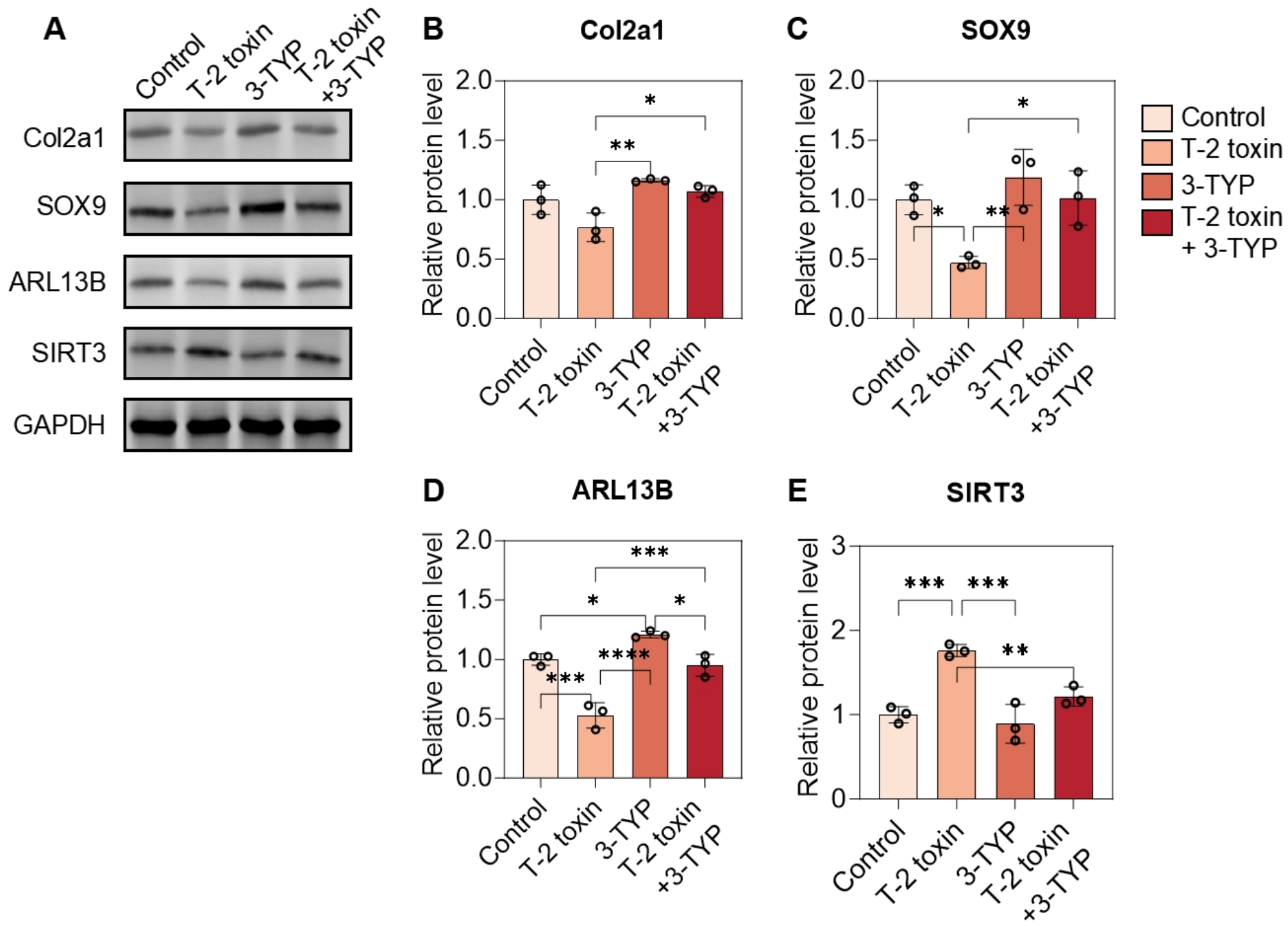

Figure S5. **(A)** Representative western blot bands. Quantitative data of **(B)** Col2a1, **(C)** SOX9, **(D)** ARL13B, and **(E)** SIRT3 protein level in chondrocytes treated with or without T-2 toxin and/or 3-TYP. N ≥ 3, n = 3. Data were compared by non-parametric one-way ANOVA followed by Tukey's multiple comparison test: * $p < 0.05$, ** $p < 0.01$, *** $p < 0.001$, **** $p < 0.0001$.

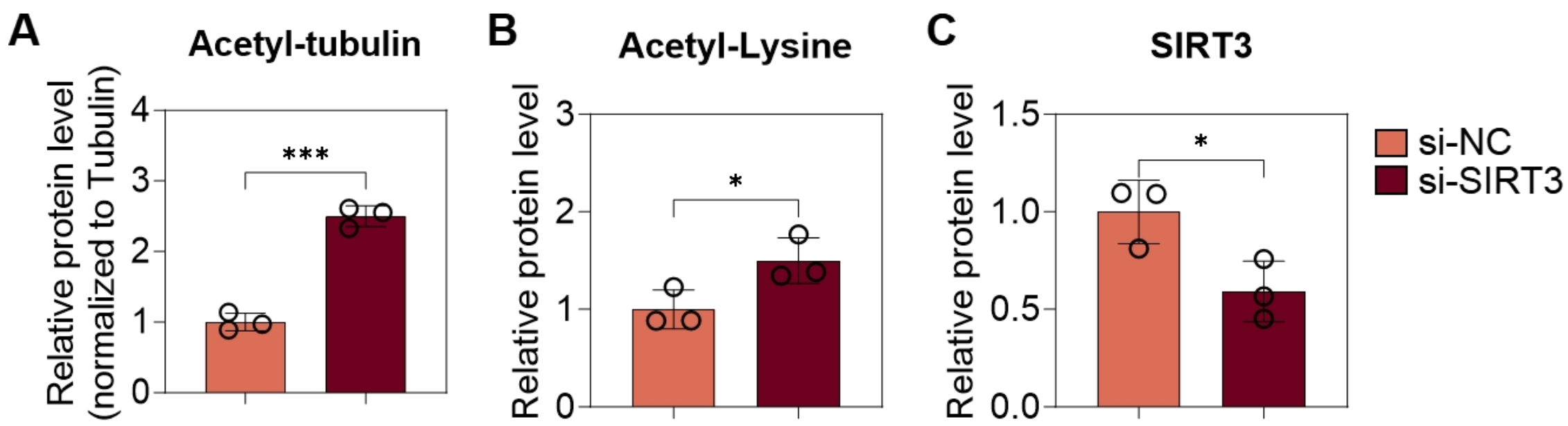

Figure S6. Quantitative data of **(A)** acetyl-tubulin, **(B)** acetyl-lysine, and **(C)** SIRT3 protein level in chondrocytes treated with si-NC or si-SIRT3. N ≥ 3, n = 3. Data were compared by unpaired, two-tailed t-test: * $p < 0.05$, *** $p < 0.001$.

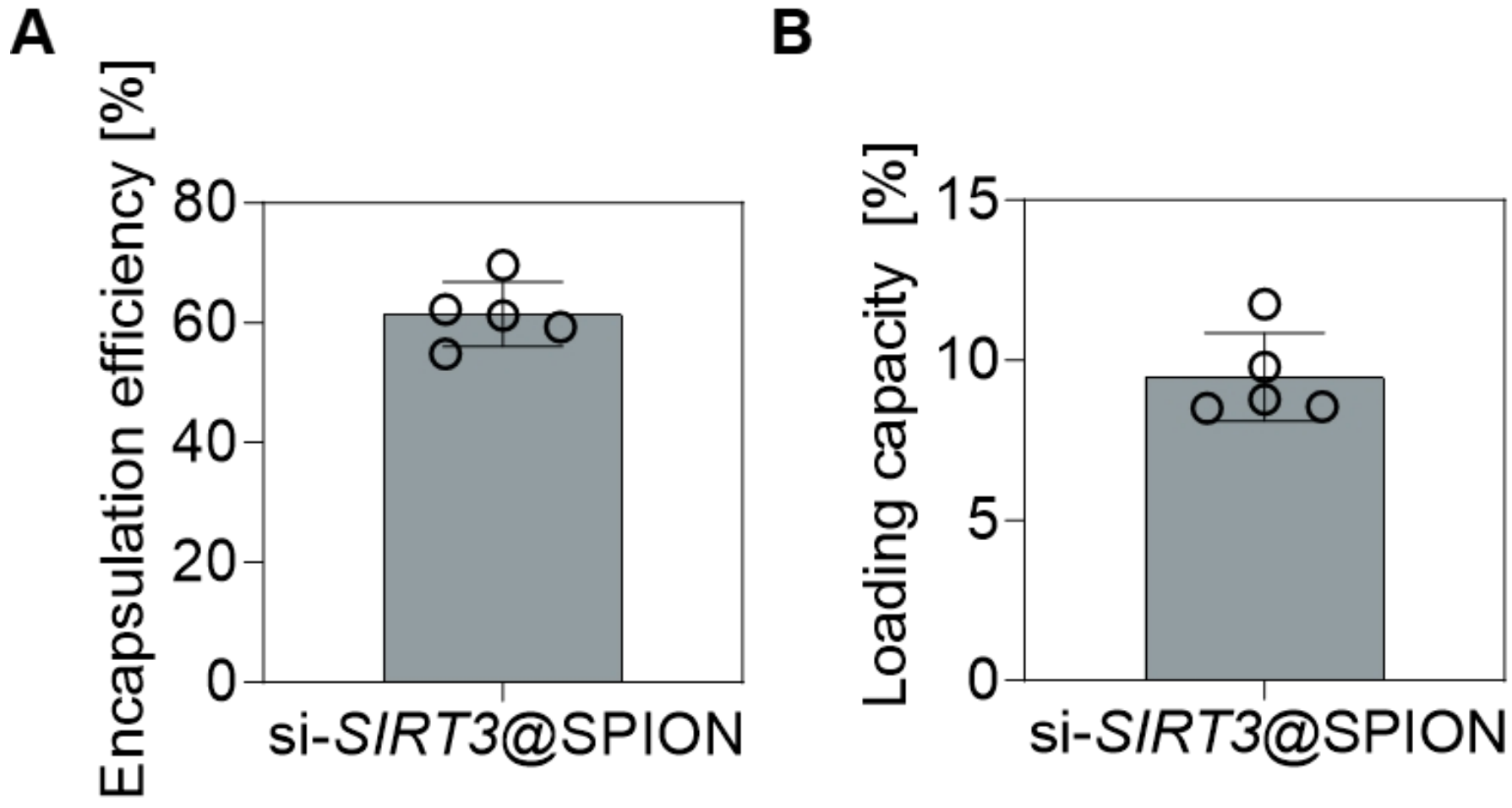

Figure S7. (A) The encapsulation efficiency (EE%) and **(B)** loading capacity (LC%) of si-SIRT3@SPIONs.

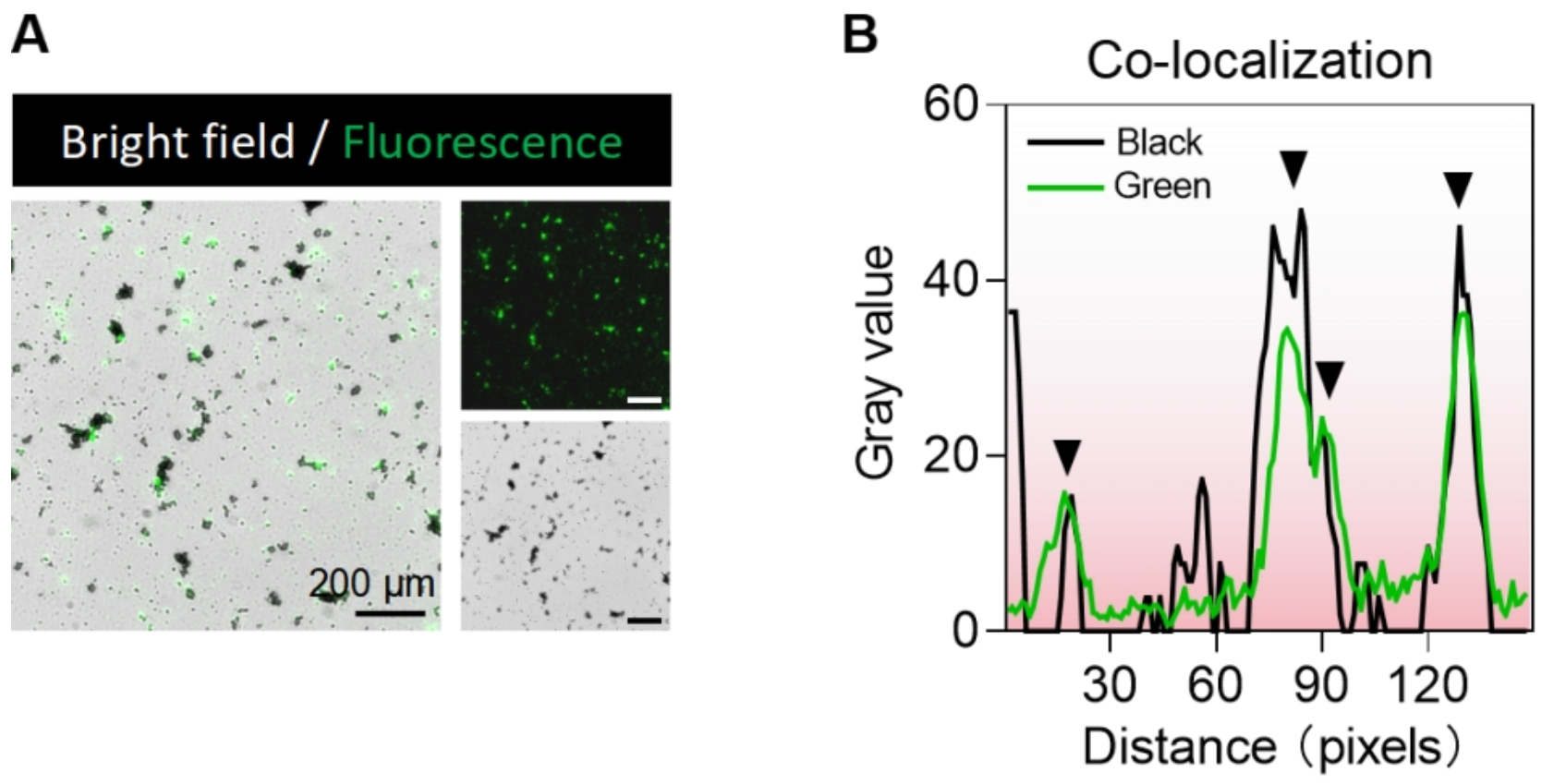

Figure S8. (A) The representative images of the FAM-labeled siRNA@SPIONs, and the **(B)** co-localization analysis.

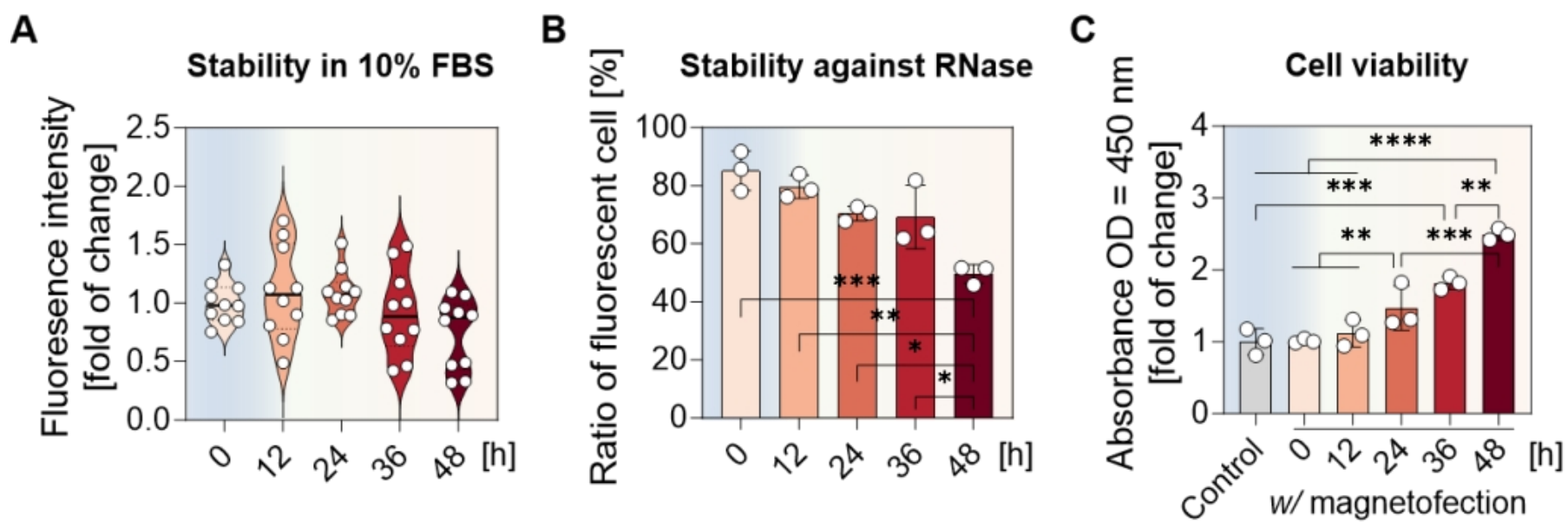

Figure S9. The stability of the si-SIRT3@SPION complex at specific time points after cell attachment. **(A)** The stability of nanocomplex in culture medium containing 10% FBS; **(B)** The stability of nanocomplex against RNase in primary chondrocytes; **(C)** Cell viability of primary chondrocytes transfected with si-SIRT3@SPION complex indirectly reflect their stability in cells. N = 3, n ≥ 3. Data were compared by non-parametric one-way ANOVA followed by Tukey's multiple comparison test: * $p < 0.05$, ** $p < 0.01$, *** $p < 0.001$, **** $p < 0.0001$.

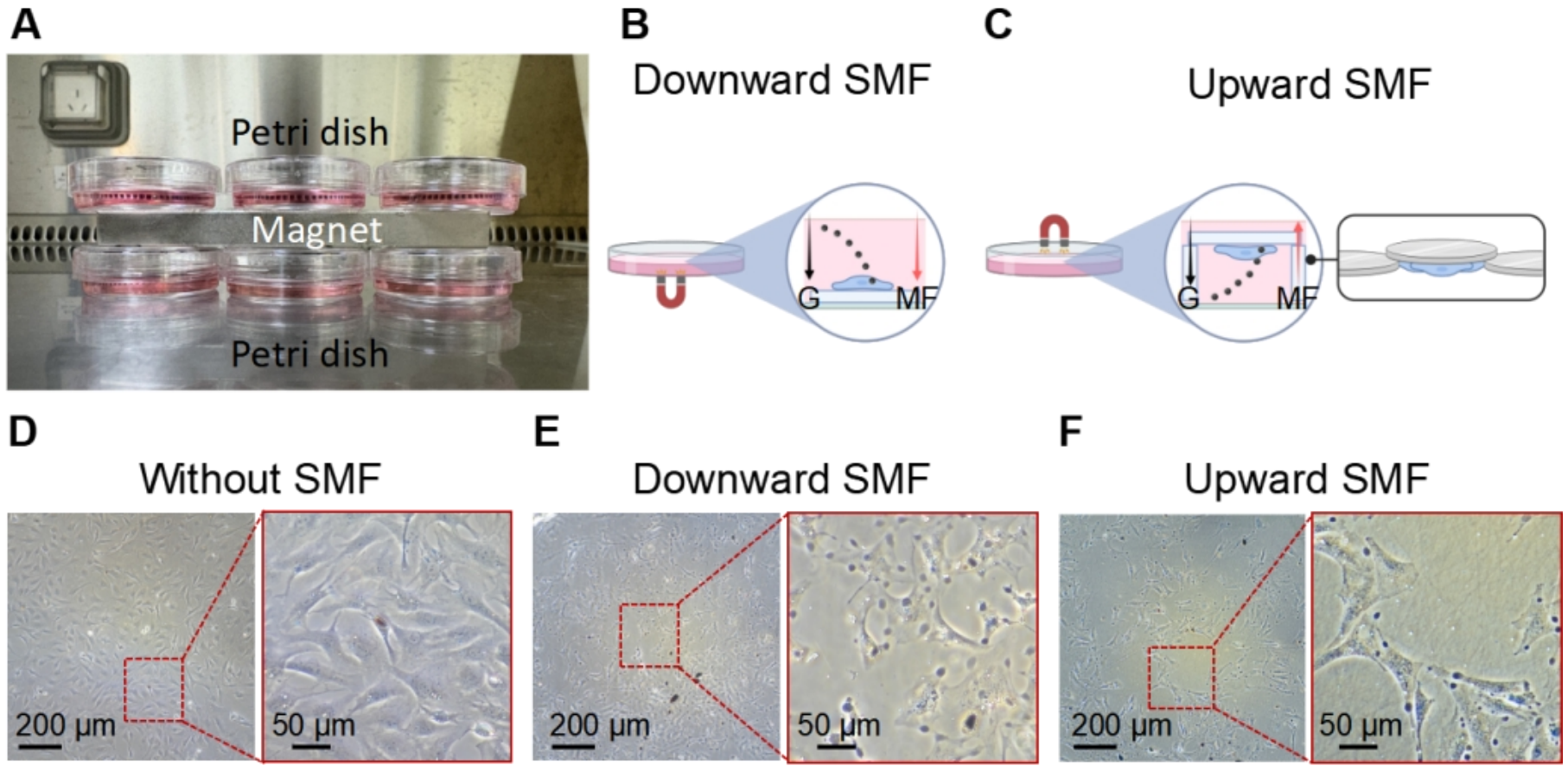

Figure S10. (A-C) The illustration of the SMF-induced manetofection. The representative images of SPIONs internalize into chondrocyte **(D)** without SMF, **(E)** with downward SMF, and **(F)** with upward SMF. Under SMF exposure, SPIONs have been internalized into chondrocytes, but SPIONs intend to agglomerate under SMF exposure.

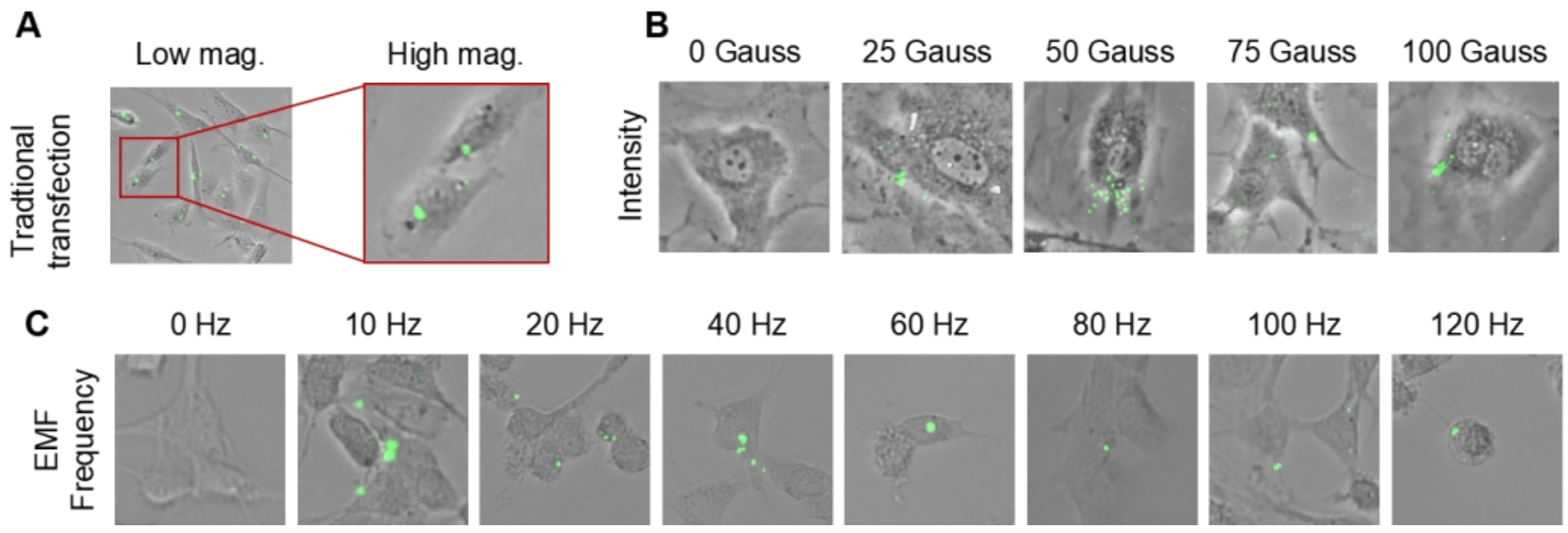

Figure S11. Fluorescent images of si-SIRT3@SPION in chondrocytes after **(A)** traditional trasfection, and EMF-induced magnetofection with different **(B)** EMF intensity, and **(C)** EMF frequency.

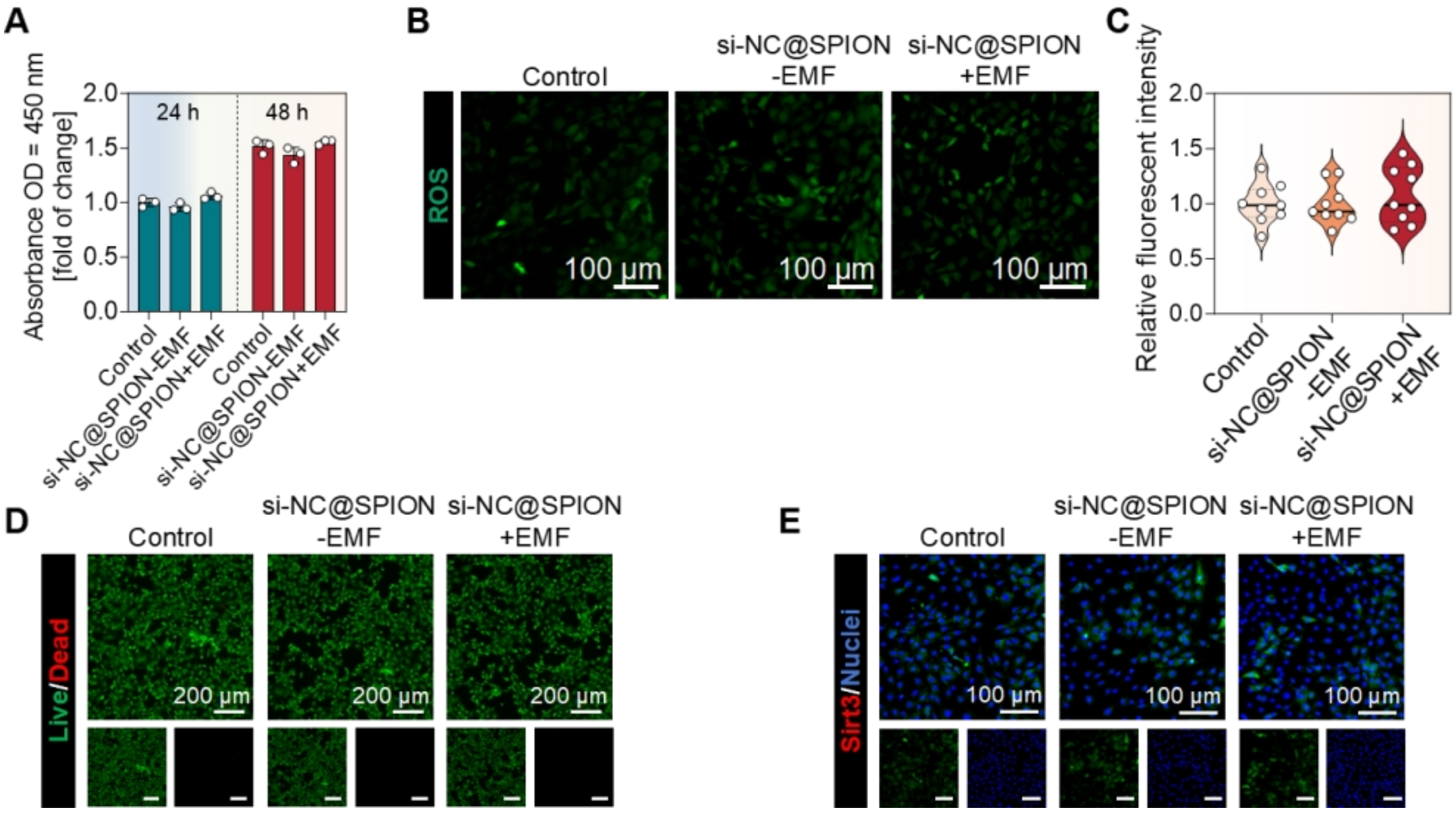

Figure S12. The effects of the si-NC@SPION on primary chondrocytes. **(A)** CCK-8 assay; **(B)** ROS staining, and **(C)** Quantitative analysis of ROS levels ; **(D)** Live/dead staining of primary chondrocytes; **(E)** Immunofluorescent staining of SIRT3 in primary chondrocytes treated with si-NC@SPION with or without EMF magnetofection. N = 3, n ≥ 3. Data were compared by non-parametric one-way ANOVA followed by Tukey's multiple comparison test.

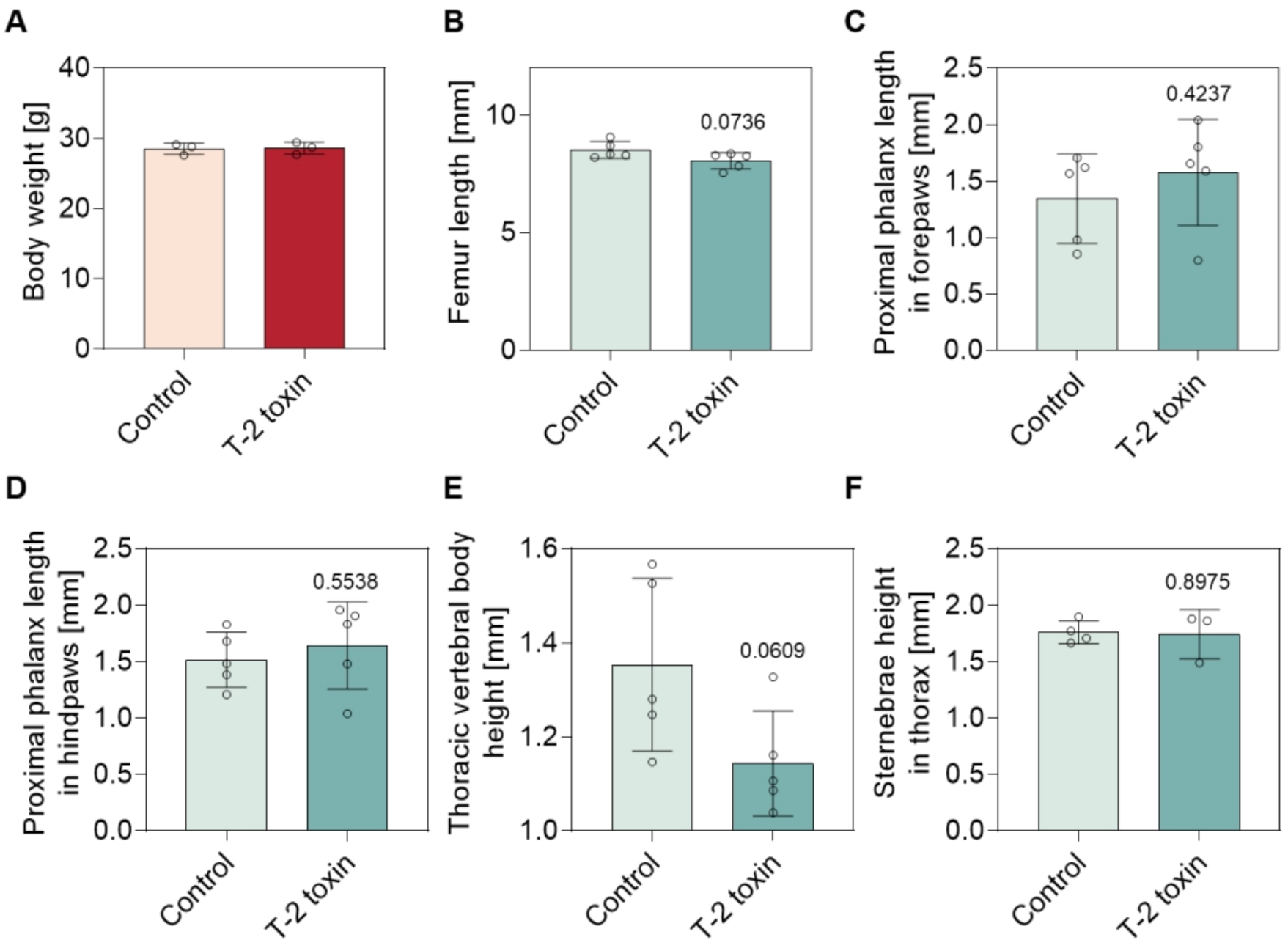

Figure S13. (A) Body weight of 10-day-old SD rats of control group or T-2 toxin group. The influences of T-2 toxin on the length of **(B)** Femur; **(C)** Proximal phalanx length in forepaws; **(D)** Proximal phalanx length in hindpaws; **(E)** Thoracic vertebral body hight; and **(F)** Sternebrae height in thorax. $N \geq 3$, $n = 3$. Data were compared by unpaired two-tailed t-test.

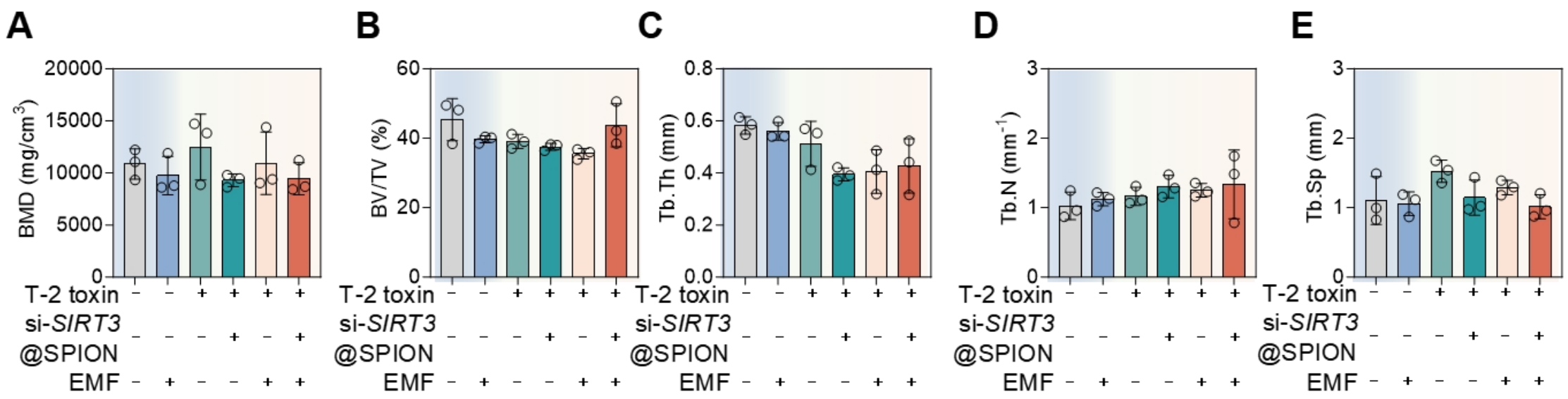

Figure S14. The quantitative analysis of bone. **(A)** BMD, **(B)** BV/TV%, **(C)** Tb.Th, **(D)** Tb.N, and **(E)** Tb.Sp. N = 3, n = 3. Data were compared by non-parametric one-way ANOVA followed by Tukey's multiple comparison test.

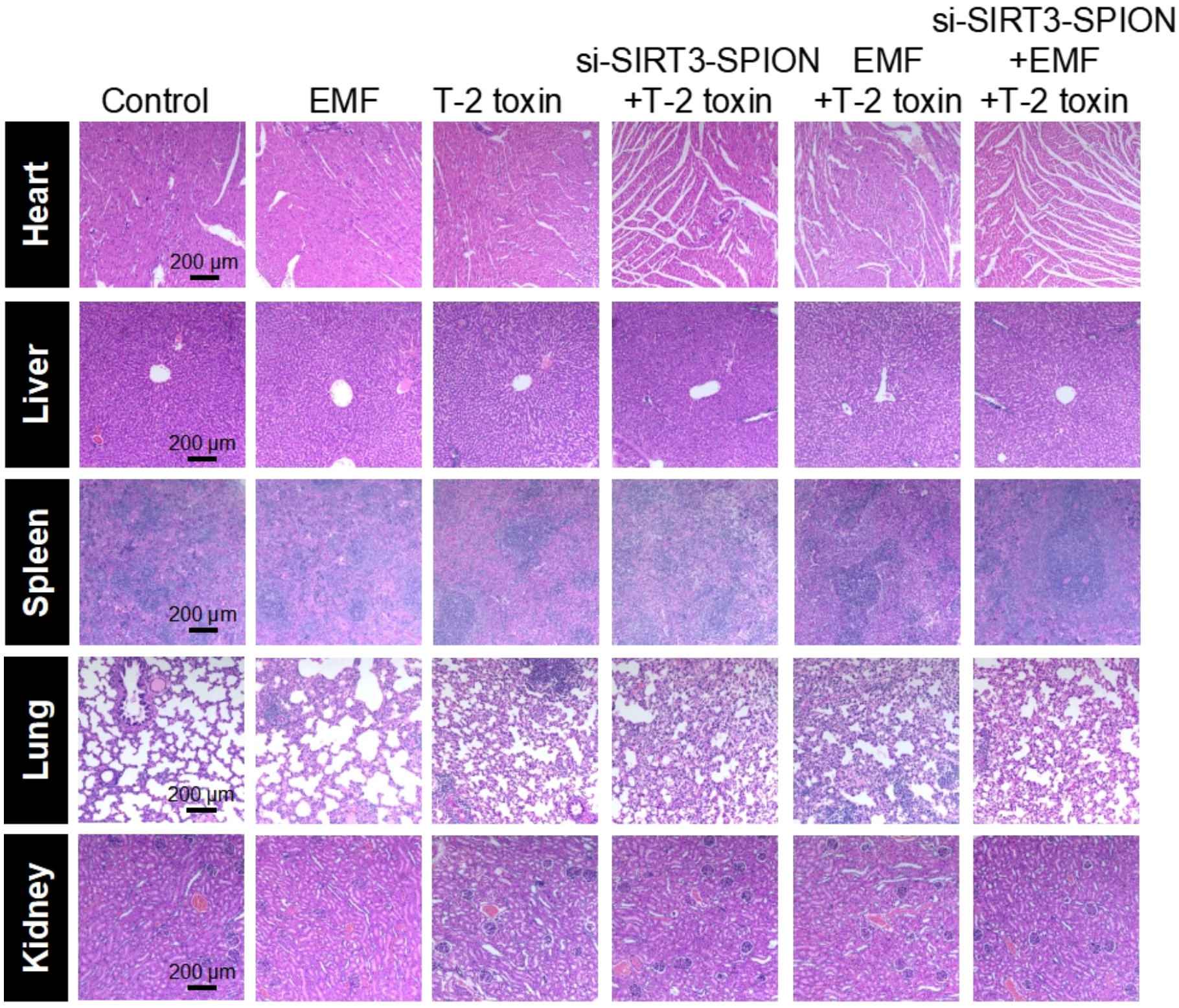

Figure S15. H&E staining of heart, liver, spleen, lung, kidney was performed to evaluate the systemic cytotoxicity.

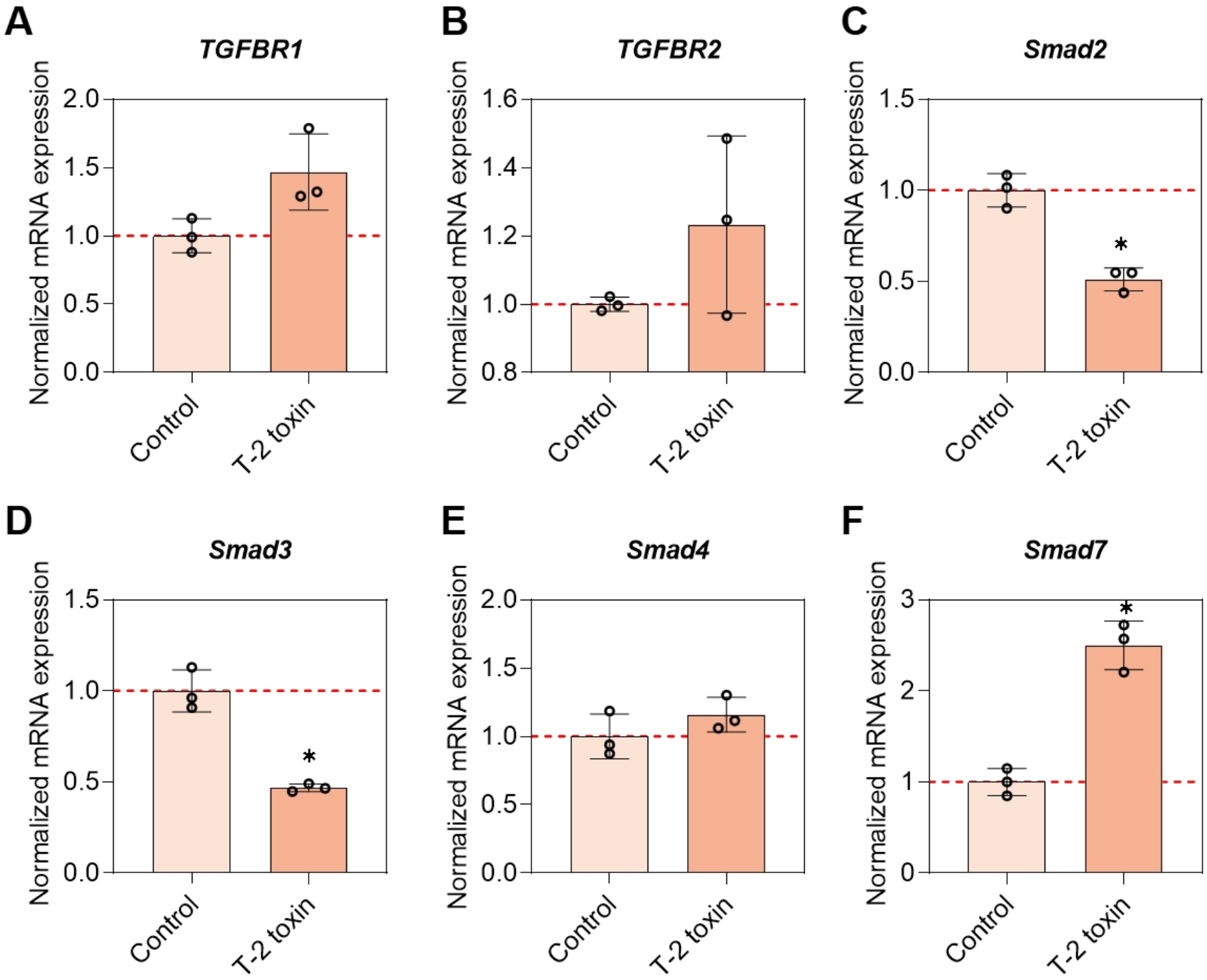

Figure S16. Influences T-2 toxin on the gene expression of **(A)** *TGFBR1*; **(B)** *TGFBR2*; **(C)** *Smad2*; **(D)** *Smad3*; **(E)** *Smad4*; and **(F)** *Smad7* in chondrocytes. N = 3, n = 3. Data were compared by unpaired two-tailed t-test: * $p < 0.05$.

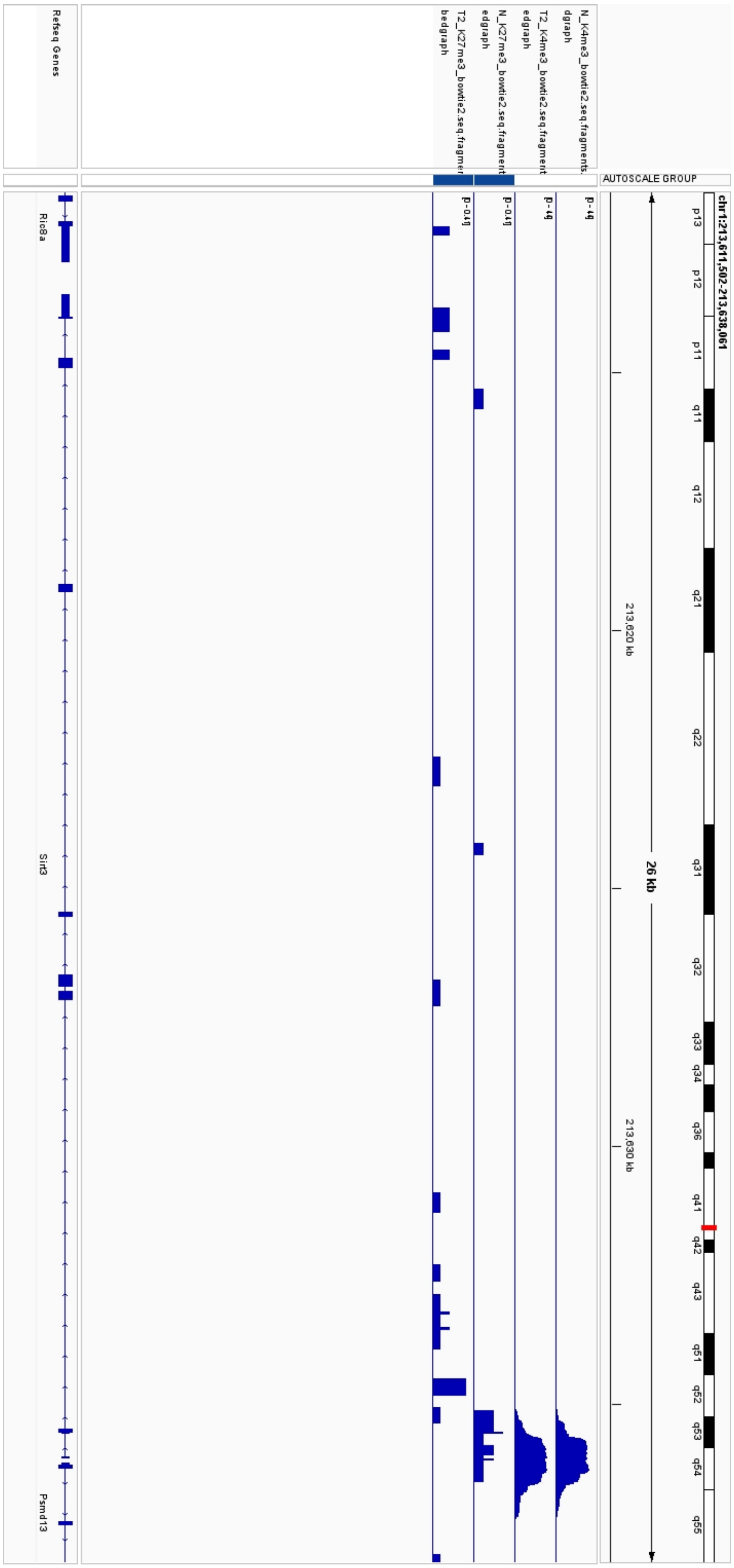

Figure S17. The sequencing data of histone methylation indicated that T-2 toxin treatment has no effects on the histone methylation of K27me3 or K4me3.

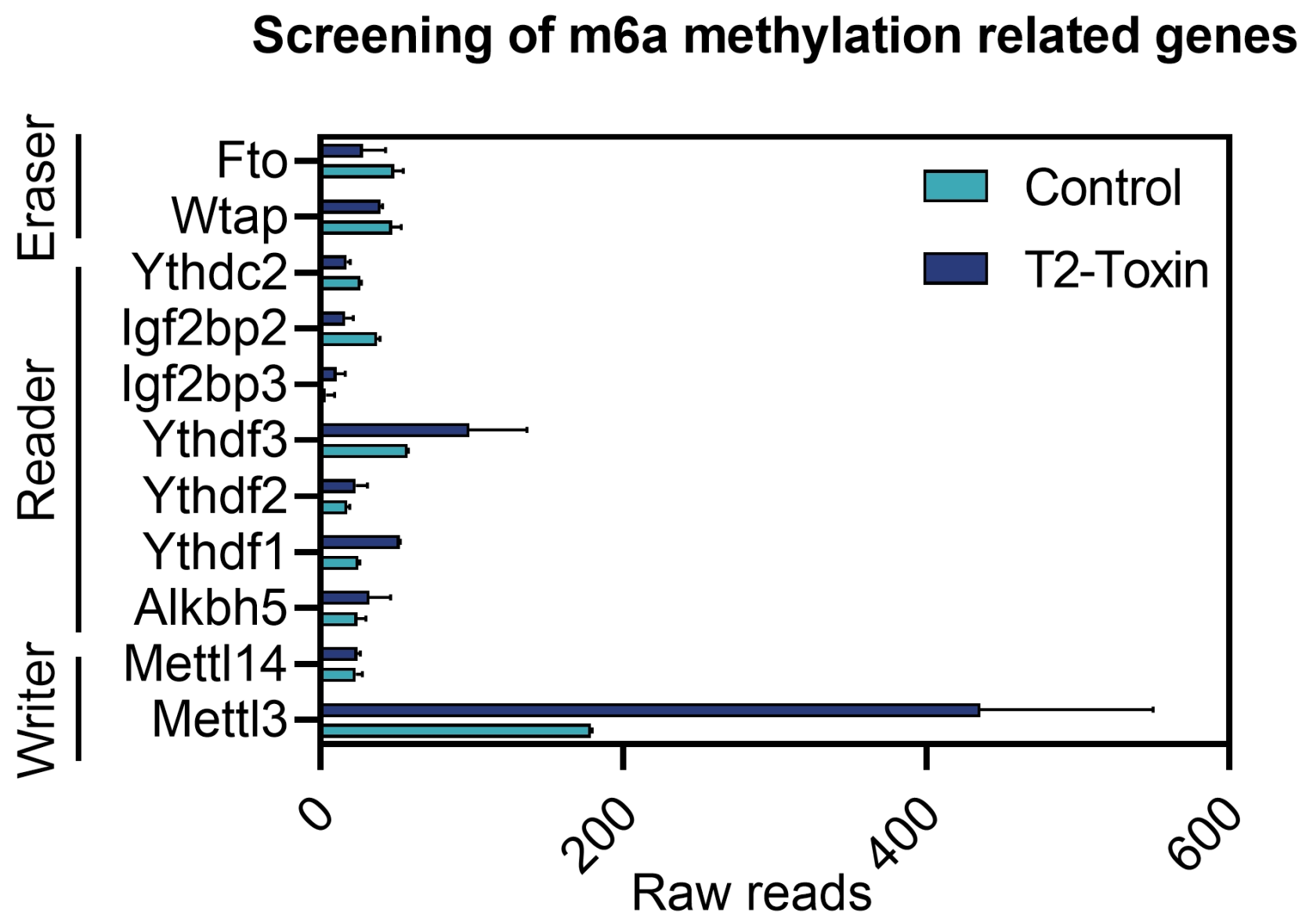

Figure S18. The screening of m6A methylation related gene, which indicated Mettl3 as the differentially expressed gene.

A

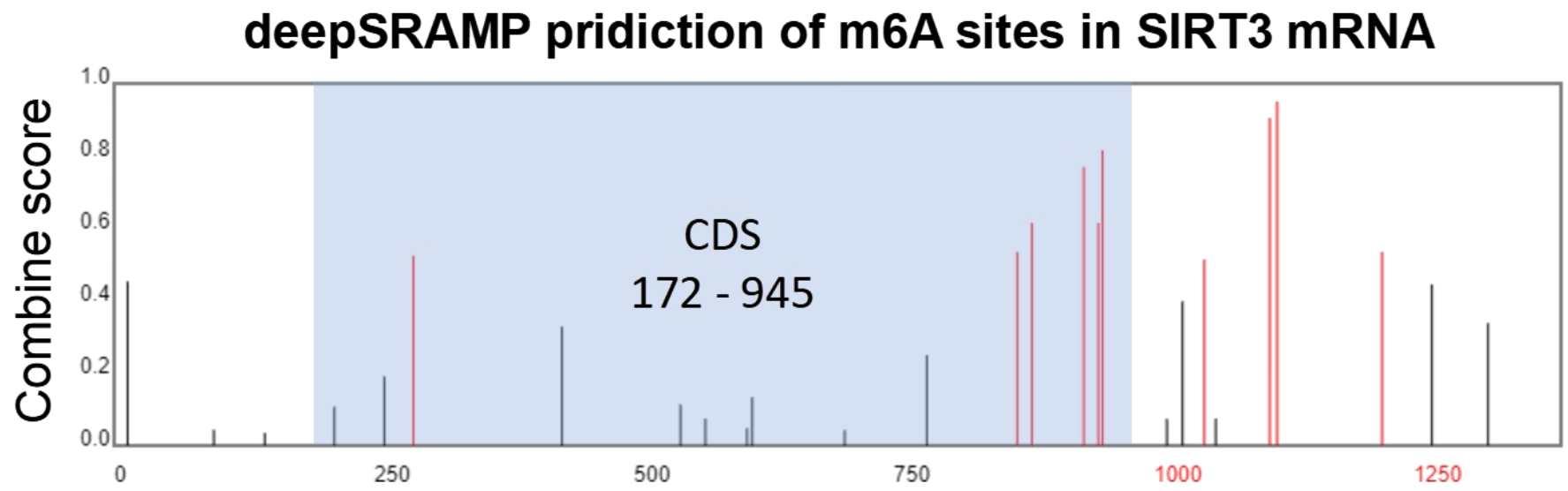

B

Data of predicted m6A sites in SIRT3 mRNA

No.	Position	Query Position	Reference Sequence Position	Motif	Probability
6	1049	1048	1048	AAACA	0.512602031
7	288	287	287	GAACT	0.523753583
10	933	932	932	GGACA	0.767356932
13	947	946	946	AGACA	0.614915669
14	951	950	950	AGACT	0.815339863
17	1220	1219	1219	TGACA	0.533966482
19	1112	1111	1111	TGACC	0.903571725
20	1119	1118	1118	GGACC	0.950102747
23	869	868	868	GGACC	0.533639848
25	883	882	882	GGACA	0.614282131

Figure S19. (A) The prediction of m6A sites of SIRT3 RNA with deepSRAMP; **(B)** The data of predicted m6A sites in SIRT3 mRNA

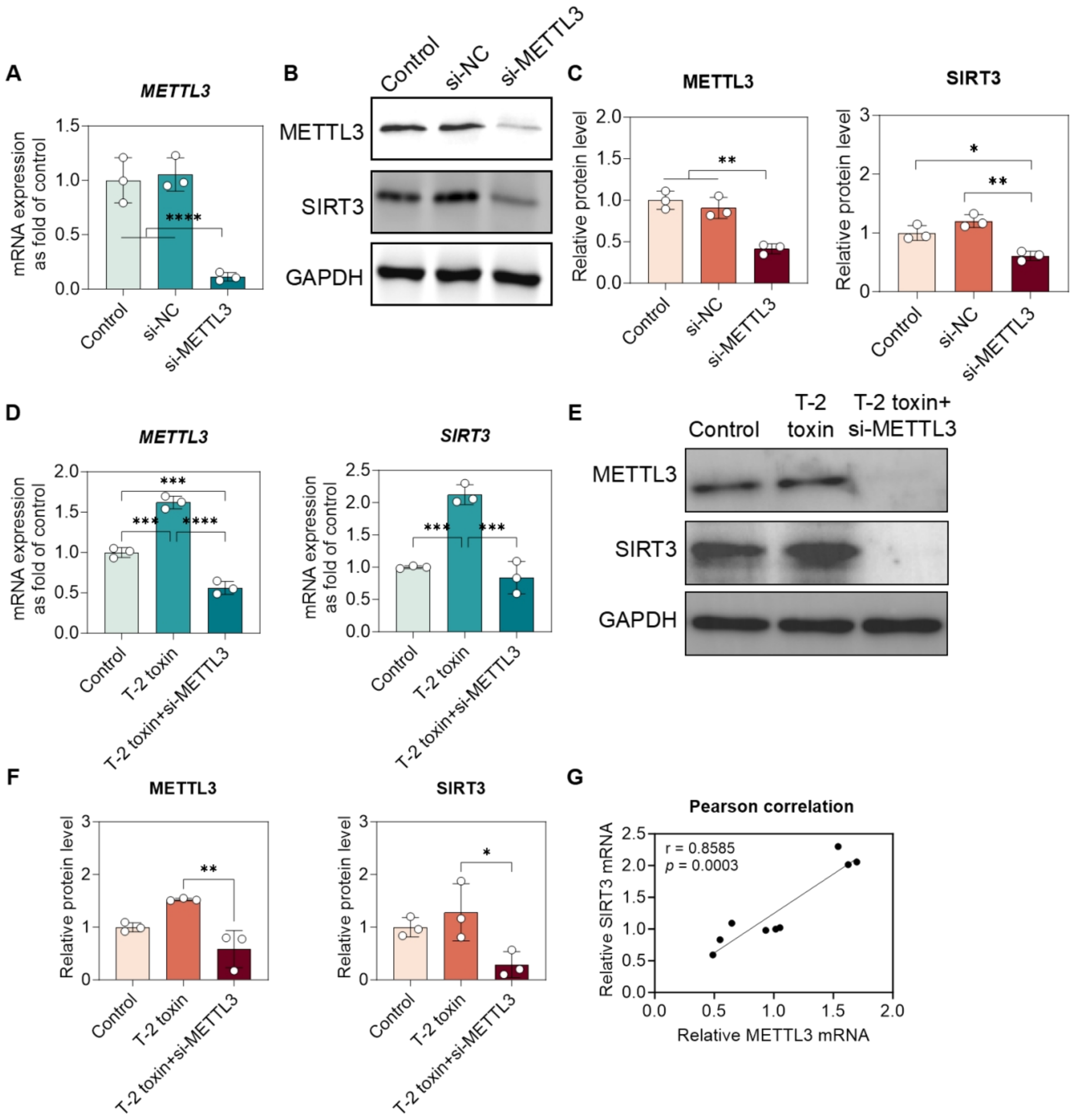

Figure S20. si-METTL3 reduced SIRT3 increased by T-2 toxin. **(A)** RT-qPCR analysis of mRNA expression of METTL3 indicated the efficiency of si-METTL3; **(B)** Representative western blot bands of METTL3, SIRT3 and GAPDH; **(C)** Quantitative analysis of METTL3 and SIRT3, which showed si-METTL3 significantly reduced the protein levels of METTL3 and SIRT3; **(D)** RT-qPCR analysis of mRNA expression of METTL3 and SIRT3 was increased with T-2 toxin treatment and reduced with si-METTL3; **(E)** Representative western blot bands of METTL3, SIRT3 and GAPDH; **(F)** Quantitative analysis of METTL3 and SIRT3, which showed si-METTL3 significantly reduced the protein levels of METTL3 and SIRT3 increased by T-2 toxin treatment; **(G)** Pearson correlation analysis was used to analyze the correlation between METTL3 and SIRT3 expression.

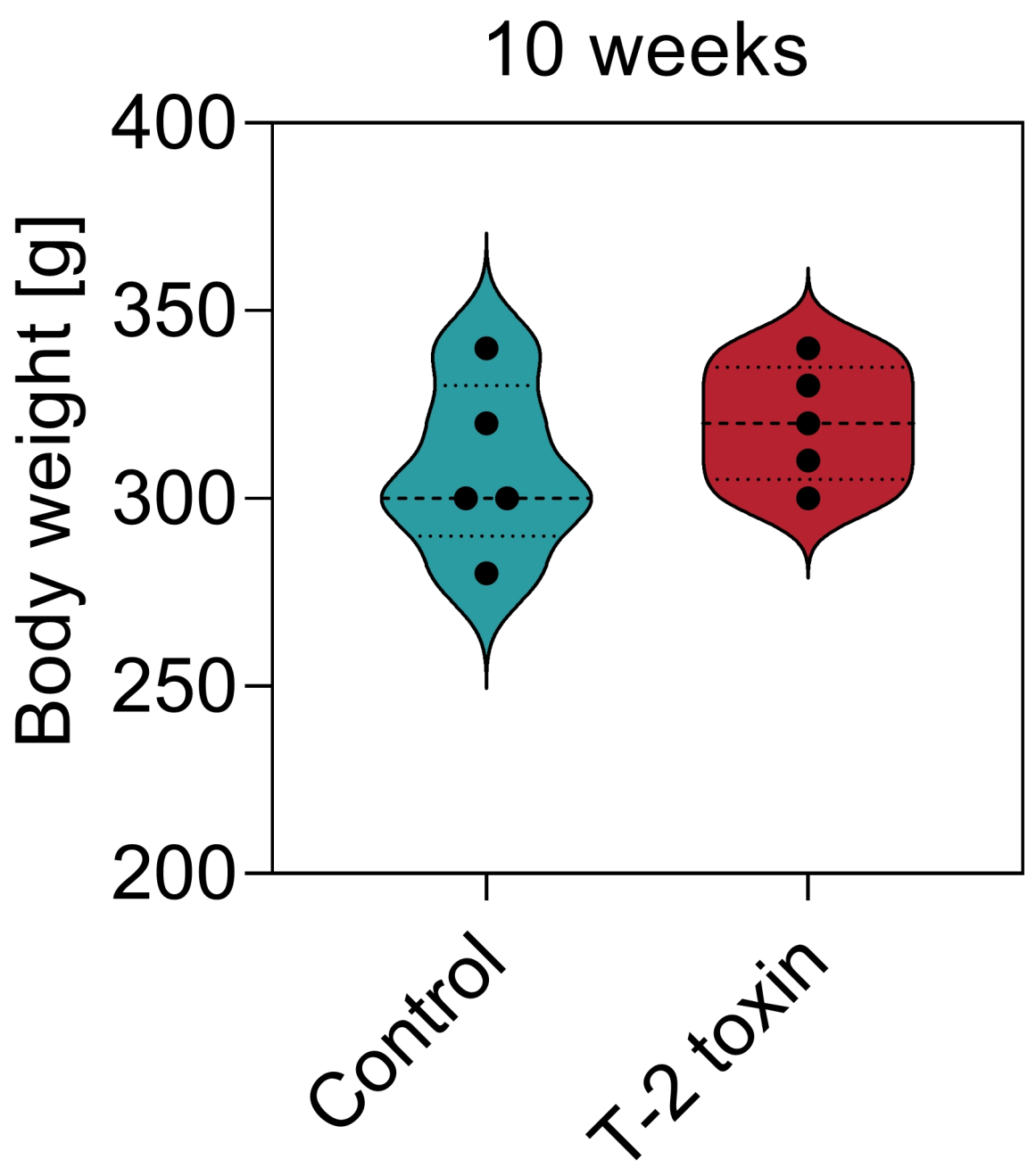

Figure S21. The body weight of 10-weeks old SD rats in control and T-2 toxin group. N ≥ 3, n = 3. Data were compared by unpaired two-tailed t-test.

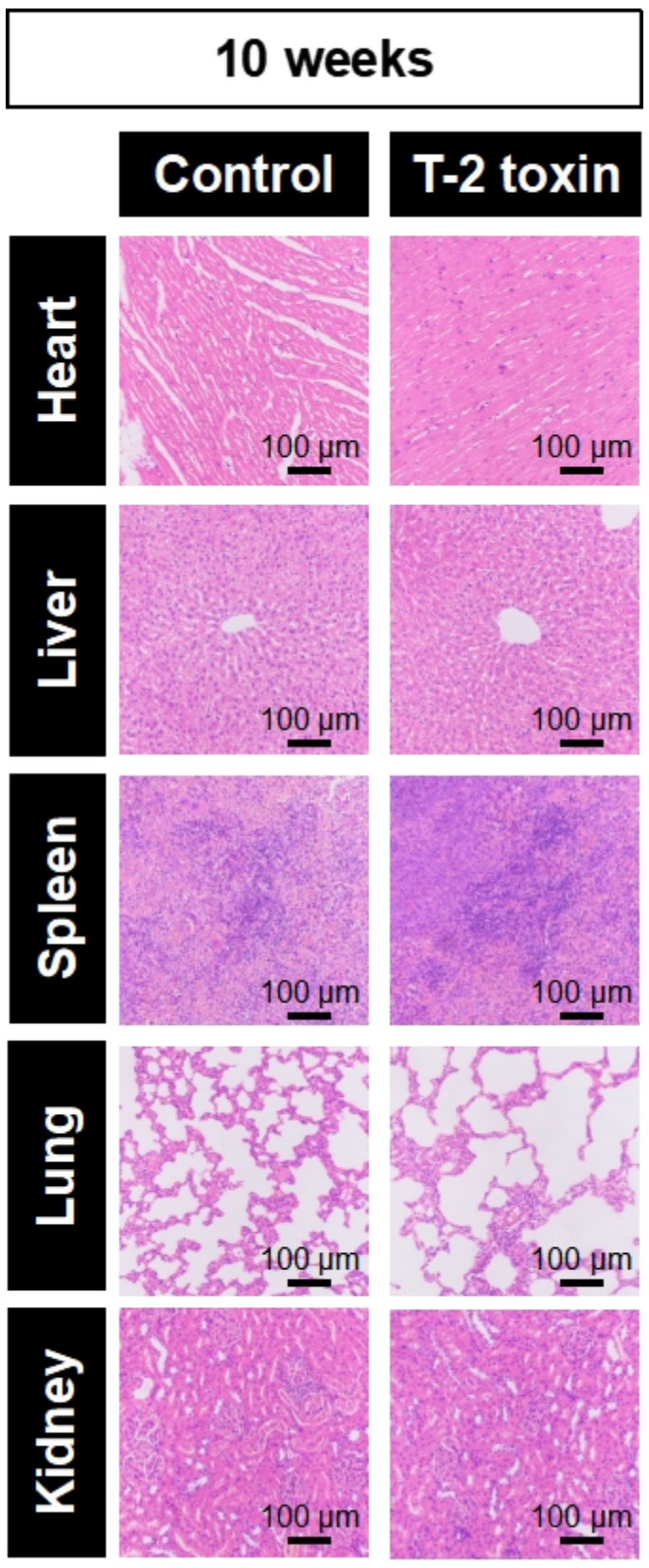

Figure S22. The H&E staining of heart, liver, spleen, lung, kidney of 10-weeks old SD rats in control and T-2 toxin group.

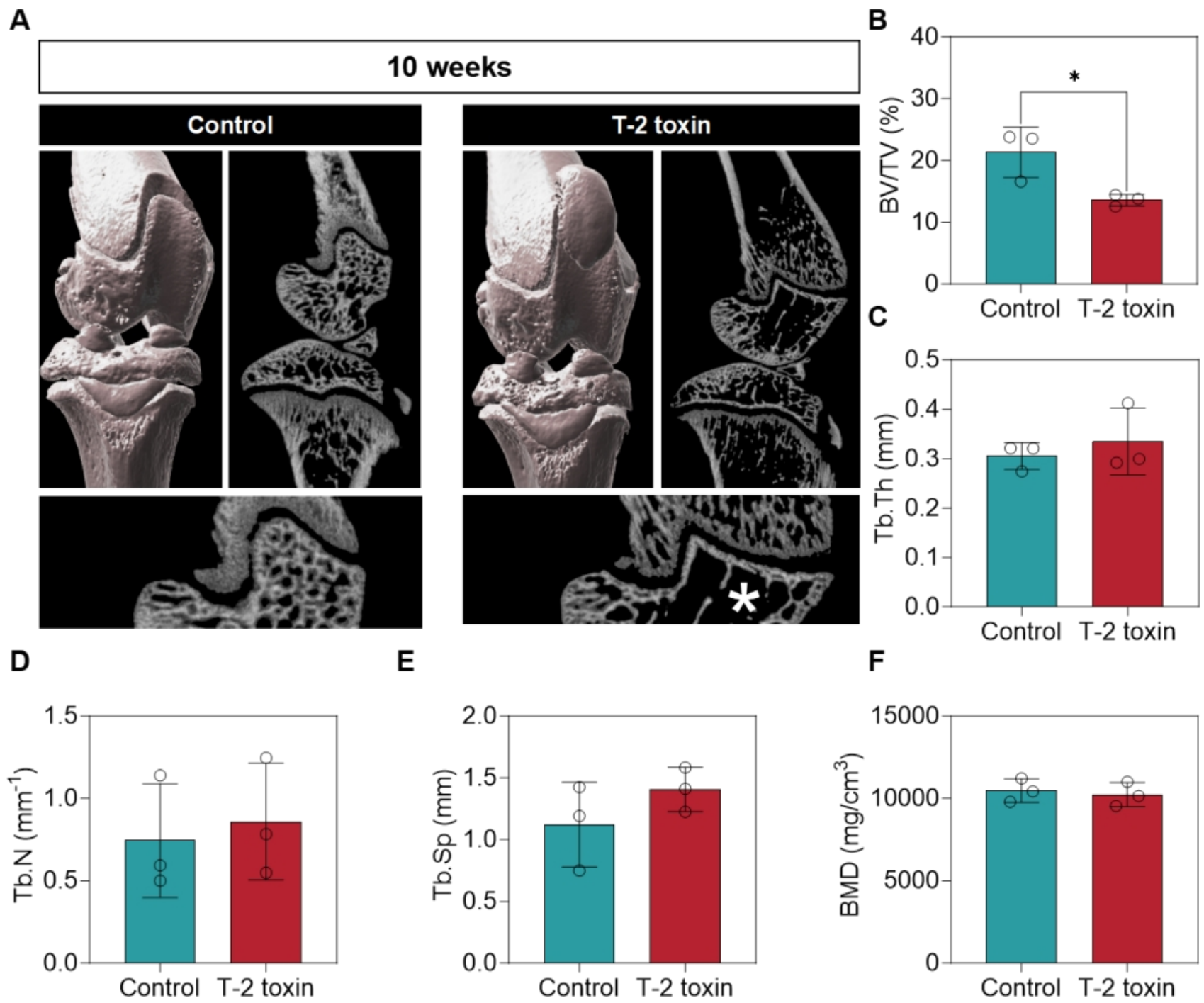

Figure S23. (A) The micro-CT images indicated the destruction of bone microstructure (indicated with a white asterisk) of knee joint of 10-weeks-old SD rats in T-2 toxin group; **(B-F)** The data analysis of the micro-CT analysis. N = 3, n = 3. Data were compared by unpaired two-tailed t-test: * $p < 0.05$.
